# Treatment of obstructive sleep apnea with a simple CPAP device

**DOI:** 10.1007/s11325-023-02823-2

**Published:** 2023-05-22

**Authors:** Zhi-Hui Qiu, Shan-Feng Liang, Xiao-Bin Deng, Qi-Shan Wei, Ying-Mei Luo, Lu Wang, Ying-Xin Wu, Joerg Steier, R. D. McEvoy, Yuan-Ming Luo

**Affiliations:** 1 State Key Laboratory of Respiratory Disease, National Clinical Research Center for Respiratory Disease, Guangzhou Institute of Respiratory Health, The First Affiliated Hospital of Guangzhou Medical University, Guangzhou, China; 2 Guangdong Medical Devices Quality Surveillance and Test Institute, Guangzhou, China; 3 Guy’s & St Thomas’ NHS Foundation Trust, King’s College London, London, UK; 4 Adelaide Institute for Sleep Health, College of Medicine and Public Health, Flinders University, Adelaide, Australia; 5 Division of Sleep Medicine, Harvard Medical School, Boston, USA

**Keywords:** OSA, Pressure titration, Automatic CPAP, Fixed CPAP

## Abstract

**Purpose:**

CPAP is the “gold standard” treatment for obstructive sleep apnea (OSA). Current CPAP models have developed additional functions including automatic CPAP and pressure relief. However, CPAP adherence has not improved over the last three decades. Many patients in low-income countries cannot afford these CPAP devices. A novel simple CPAP device with a fixed pressure without pressure controller was developed.

**Methods:**

Manual CPAP pressure titration was performed in 127 patients with OSA. Six patients with a titration pressure higher than 11 cmH_2_O and 14 patients who could not tolerate CPAP were excluded, leaving 107 participating in the following 2 studies. In study one, 54 of 107 patients were treated by both conventional fixed CPAP and simple CPAP in random order. In the second study, another 53 patients were treated by both autoCPAP in automatic function and simple CPAP in random order. Simple CPAP was fixed at 10 cmH_2_O, 8 cmH_2_O, and 6 cmH_2_O for patients whose titration pressure was between 9–10, 7–8, and ≤ 6 cmH_2_O, respectively. Conventional fixed CPAP device was set exactly the same as manual titration pressure.

**Results:**

All patients whose manual titration pressure ≤ 10 cmH_2_O were effectively treated by simple CPAP (AHI 40.7 ± 2.3 events/h before vs 2.5 ± 0.3 events/h after, *p* < 0.001). Patients expressed similar preferences for simple CPAP, autoCPAP, and conventional fixed CPAP (*p* > 0.05).

**Conclusions:**

We conclude that a novel simple CPAP is an alternative treatment for most patients with OSA, which may widen access to CPAP therapy in the developing countries because of its low cost.

**Supplementary Information:**

The online version contains supplementary material available at 10.1007/s11325-023-02823-2.

## Introduction

Obstructive sleep apnea (OSA) is a common disease. Moderate to severe OSA defined as an apnea–hypopnea index (AHI) of more than 15 events/h affects between 6 and 17% of the general population [1]. The prevalence of OSA has continuously increased, mainly because of the increasing prevalence of obesity [2], although increased life expectancy [3] may also be pertinent because age is a risk factor for OSA [4, 5].

The major physiological abnormalities in OSA are repetitive upper airway occlusions that result in intermittent hypoxia, increased inspiratory pressure swings, and frequent arousals from sleep. Approximately half of individuals affected experience daytime sleepiness that contributes to an increased risk of road traffic accidents, cardiovascular events, depression, and diabetes [6].

Effective intervention, using nasal continuous positive airway pressure (CPAP), can significantly improve OSA-related outcomes including daytime sleepiness, quality of life, and insulin resistance, although outcomes for cardiovascular disease may not necessarily be improved [7]. One of the challenges of OSA treatment is that many patients cannot afford CPAP if the costs need to be covered by themselves [8, 9]. Unsurprisingly, this problem is most acute in low-income countries [8, 10]. In these countries, more than 50% of patients with severe OSA may not be able to afford to buy a CPAP device. This impediment to OSA therapy may be not confined to low-income countries. For example, financial issue related to CPAP therapy has also been reported among low-income residents of high-income countries [9]. Developing an effective simple CPAP device is essential to enhance uptake of this therapy globally [11].

One reason that current CPAP devices may be costly is that current models offer multiple additional functions such as remote telemonitoring interactions, leak measurement, bi-level and variable level pressure, pressure relief during expiration, and ramping of pressure, as well as significant data storage and processing functions. Interestingly, despite these additional functions being added to CPAP devices, acceptance and adherence to CPAP or the efficacy of treatment has remained largely unchanged since its invention in the 1980s [12, 13]. This suggests that the additional functions added to CPAP devices do not materially improve patient comfort or treatment efficacy.

The main principle of CPAP therapy in patients with OSA is to maintain upper airway patency with positive airway pressure while asleep. Standard CPAP devices can be adjusted to any pressure level between 4 and 20 cmH_2_O, but the controller hardware and associated software needed to make this adjustment significantly contribute to the cost of modern CPAP devices. However, based on manual titration studies done by us [14] and others [15, 16], the CPAP pressure required for the effective treatment in the majority of patients is less than or equal to 10 cmH_2_O.

We therefore developed a simple CPAP with the aim of reducing the cost of CPAP. Our simple CPAP device has no pressure controller components and no additional functions such as ramp, pressure relief, or automatic change in pressure, and it only requires a power switch for full operation. The pressures delivered by the simple CPAP device were pre-set in the factory to a default at 10, 8, or 6 cmH_2_O. In the current study, we compared this device with conventional fixed pressure CPAP and automatic CPAP (autoCPAP) devices to determine whether or not a simple CPAP could effectively treat patients with OSA.

## Methods

The study was approved by the ethics committee of the First Affiliated Hospital of Guangzhou Medical University, China, and was registered on ClinicalTrials.Gov (registration no. NCT03782844). A written informed consent was obtained from all the participants.

### Patient selection

**Fig. 1:**
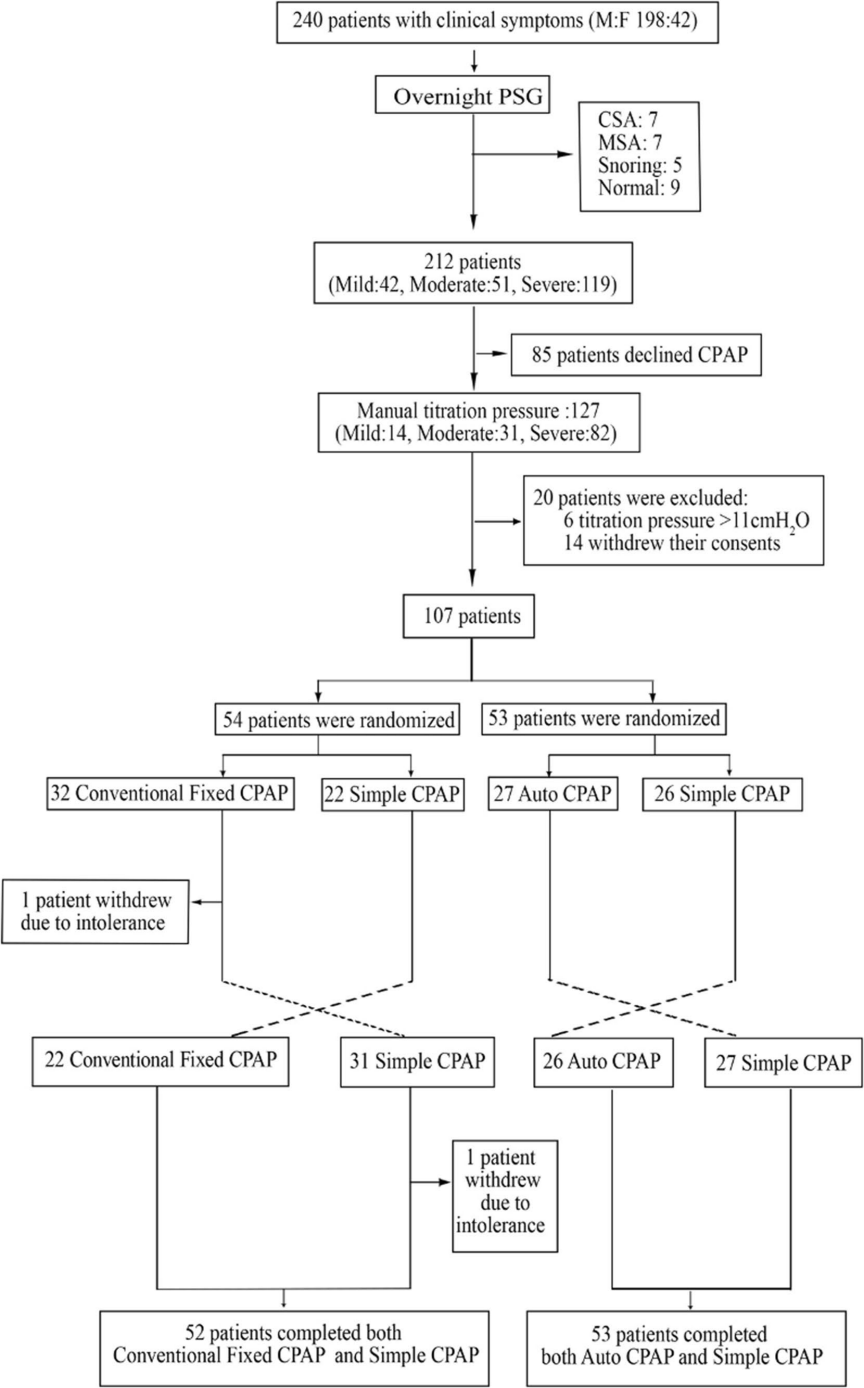
Flow of patient recruitment

Patient recruitment flow is shown in Fig. 1. Briefly, 240 patients (aged 48 ± 0.9 year) with daytime sleepiness or snoring visiting outpatient clinic between June 2017 and January 2019 were referred for overnight full polysomnography at the Sleep Center of the First Affiliated Hospital of Guangzhou Medical University, Guangzhou, China, for clinical reasons. Patients with obstructive pulmonary disease, severe neuromuscular disease, and respiratory failure were excluded.

Of these, 212 patients were diagnosed with OSA based on polysomnography using a criterion of AHI ≥ 5 events/h and deemed to require CPAP therapy. These patients were invited to participate in the study. Eighty-five of 212 patients declined to accept CPAP therapy, leaving 127 patients with OSA who proceeded to have a second night sleep study in the sleep laboratory during which manual titration was undertaken to identify the optimal CPAP pressure.

Of the 127, fourteen patients could not tolerate CPAP and withdrew their consent from the study during pressure titration, and 6 further patients whose titration pressure was higher than 11 cmH_2_O were excluded for further comparison between simple CPAP and conventional CPAP or autoCPAP.

Consequently, 107 patients were randomized to compare simple CPAP with conventional fixed pressure CPAP (study 1, *n* = 54) or autoCPAP (study 2, *n* = 53). To avoid bias during the study, all devices for comparison (simple CPAP, conventional fixed CPAP, and autoCPAP) were sealed inside an unlabeled box. Patients did not know the device with which they were treated. The mask and tubing used for the study were exactly the same for each patient. At the end of the study, patients were asked for their preference as to which device they preferred for the future treatment.

### Polysomnography

Full-attended polysomnography (Alice 5, Philips Respironics, USA) was performed in all cases. Two channels of electroencephalogram (C4A1, C3A2), left and right electrooculograms (EOGs), submental EMG (EMGchin), airflow from both thermistor and nasal pressure, chest and abdominal movements, as measured by uncalibrated respiratory inductance plethysmography (RIP), arterial oxygen saturation (SaO2), and body position were recorded. In the first (diagnostic) night of polysomnography, we also recorded the Epworth sleepiness scale (ESS) score and demographic data including height, weight, and neck and waist circumference.

In the second and therapeutic night, CPAP was manually titrated with a device (REMstar Auto, CFlex, Philips Respironics, PA, USA) used in manual mode. Patients were again studied in the sleep laboratory monitored with full PSG (Alice 5, Philips Respironics, PA, USA) based on our standard procedures, as previously described [14]. In brief, CPAP was started at 4.0 cmH_2_O and gradually increased in steps of 1 cmH_2_O every 10 min until apneas and hypopneas were controlled. When no further respiratory events were observed for half an hour, CPAP was decreased again by 1 cmH_2_O every 10 min until apnea–hypopnea events recurred. This cycle was repeated until the optimal therapeutic pressure was obtained, defined as the lowest effective pressure for eliminating apnea and hypopnea events in all body positions and sleep stages including rapid eye movement (REM) sleep during supine position. The minimum acceptable time for this titration process was 3 h.

### Study 1: comparison between simple CPAP and conventional fixed CPAP

The first 54 of the 107 newly diagnosed patients who had completed manual titration were treated with both conventional fixed CPAP (REMstar Auto A-Flex (557P), Philips Respironics, USA) and simple CPAP (Respiratory Medical Science Ltd. Co, Guangzhou, China) on two separate nights in random order, and the effects were monitored by full polysomnography. The simple CPAP device commercially named as Sui-Kang (SK) had a default pressure at low level (6 cmH_2_O), at middle level (8 cmH_2_0), or at high level (10 cmH_2_O). For the conventional fixed CPAP treatment, pressure was set at the level the same as that derived from the manual titration. However, selection of the type of simple CPAP was based on the following criteria: if manual titration pressure was 6 cmH_2_O or less, then 6 cmH_2_O was selected; if the manual titration pressure was 7–8 cmH_2_O, 8 cmH_2_O was selected; and if the CPAP pressure derived from manual titration was 9–10 cmH_2_O, 10 cmH_2_O was selected. Two of the 54 patients dropped out from the study before completion of the comparison. Consequently, comparison between conventional fixed CPAP and simple CPAP was possible in 52 patients. All the patients used the conventional fixed CPAP treatment without the ramp, pressure release, and humidity.

### Study 2: comparison between simple CPAP and autoCPAP

The second group consisted of the subsequent 53 of the 107 patients with OSA after titration. They were treated both by autoCPAP (REMstar Auto, A-Flex, 557P, Philips Respironics, PA, USA) in automatic function and simple CPAP in random order on two different nights within a week. The pressure for the simple CPAP device was selected based on the criteria, as described above (see study 1). The autoCPAP was set in automatic mode with the permit pressures between 4 and 20 cmH_2_O. Additional functions such as pressure release (A-flex level 3), humidity, and pressure ramp were on.

### Data analysis

Data obtained from overnight full polysomnography were analyzed based on American Academy of Sleep Medicine (AASM) criteria 2012 [17] for scoring respiratory events by a qualified technician with more than 10 years of experience in PSG. The technician who analyzed the data was blinded to the device allocation. AHI, 3% oxygen desaturation index (ODI), arousal events/hour (arousal index), sleep efficacy, and duration of CPAP treatment were analyzed and reported. Data analyses were performed using SPSS for Windows, version 20.0 (SPSS, Inc., Chicago, IL, USA). A paired *t*-test was used to assess the difference between different CPAP devices. Patients’ preference to CPAP model was assessed by chi-square test. All tests were considered statistically significant if *p* < 0.05.

## Results

Baseline PSG data including AHI, ODI, arousal index, sleep efficacy and participant characteristics in 127 patients are reported in Table 1 and online supplementary table E-1 and table E-2. In six patients, the effective titration pressure was above 11cmH_2_0 (three patients at 11 cmH_2_O and three others at 12 cmH_2_O). However, no patients had a manual titration pressure higher than 12 cmH_2_0 in the study. For most patients (95.3%), the effective manually titrated pressure was below 10cmH_2_O (Table 2): 48.0% with ≤ 6 cmH_2_O, 32.3% with 7–8 cmH_2_O, and 15% with 9–10 cmH_2_O.

**Table 1:** Participant characteristics (mean ± SE; *n* = 127)

Variables	Results
Age (years)	51.0 ± 1.3
Sex (male/female)	104/23
Body mass index (kg/m^2^)	27.2 ± 0.3
Neck circumference (cm)	38.7 ± 0.3
Waist circumference (cm)	96.5 ± 1.0
Hip circumference (cm)	101.3 ± 0.7
Epworth sleepiness scale score	7.2 ± 0.5
Systolic BP (mmHg)	129.5 ± 1.4
Diastolic BP (mmHg)	77.2 ± 1.1
Hypertension (*n*, %)	45 (35.4)
Coronary heart disease (*n*, %)	16 (12.6)
Stroke (*n*, %)	11 (8.7)
Diabetes (*n*, %)	13 (10.2)
Asthma (*n*, %)	1 (0.8)
COPD (*n*, %)	3 (2.4)
Current cigarette smoking (*n*, %)	59 (46.5)
Alcohol use (*n*, %)	11 (8.7)
Polysomnography	
Total record time (min)	497.5 ± 4.3
Total sleep time (min)	396.7 ± 5.9
Sleep onset latency (min)	19.7 ± 2.2
Sleep efficiency (%)	80.2 ± 1.2
Arousal index (events/h)	33.2 ± 1.8
REM sleep (%)	16.8 ± 0.6
Stage 1 (%)	24.8 ± 1.3
Stage 2 (%)	52.1 ± 1.1
Stage 3 (%)	6.2 ± 0.6
AHI (events/h)	42.5 ± 2.2
OAI (events/h)	20.5 ± 1.7
CAI (events/h)	2.3 ± 0.3
MAI (events/h)	4.8 ± 0.7
HI (events/h)	14.8 ± 1.0
Sleep time mean SaO_2_ (%)	94.0 ± 0.3
Lowest SaO_2_ (%)	75.9 ± 1.1
3% ODI (events/h)	30.6 ± 2.1

**Table 2:** Respiratory events, sleep structure during overnight titration, and the subject number at different titrated pressure (mean ± SE)

Parameter	Baseline	Manual titration	*p* value
Total record time (min)	497.5 ± 4.3	502.4 ± 4.6	0.373
Total sleep time (min)	396.7 ± 5.9	415.1 ± 6.1	0.006
Sleep onset latency (min)	19.7 ± 2.2	22.3 ± 3.1	0.491
Sleep Efficiency (%)	80.2 ± 1.2	82.4 ± 1.3	0.120
Arousal index (events/h)	33.2 ± 1.8	10.8 ± 0.4	0.000
REM sleep (%)	16.8 ± 0.6	23.2 ± 0.7	0.000
Stage 1 (%)	24.8 ± 1.3	15.4 ± 0.9	0.000
Stage 2 (%)	52.1 ± 1.1	53.0 ± 1.1	0.482
Stage 3 (%)	6.2 ± 0.6	8.4 ± 0.8	0.006
AHI (events/h)	42.5 ± 2.2	4.7 ± 0.4	0.000
OAI (events/h)	20.5 ± 1.7	0.9 ± 0.1	0.000
CAI (events/h)	2.3 ± 0.3	0.9 ± 0.1	0.000
MAI (events/h)	4.8 ± 0.7	0.1 ± 0.0	0.000
HI (events/h)	14.8 ± 1.0	2.8 ± 0.2	0.000
Sleep time mean SaO_2_ (%)	94.0 ± 0.3	96.2 ± 0.1	0.000
Lowest SaO_2_ (%)	75.9 ± 1.1	88.0 ± 0.9	0.000
ODI (events/h)	30.6 ± 2.1	3.6 ± 0.3	0.000
Pressure ≤ 6 cmH2O (*n*, %)		61 (48.0%)	
Pressure 7–8 cmH2O (*n*, %)		41(32.3%)	
Pressure 9–10 cmH2O (*n*, %)		19 (15.0%)	
Pressure ≥ 11 cmH2O		6 (4.7%)	

### Manual titration vs automatic titration

**Fig. 2:**
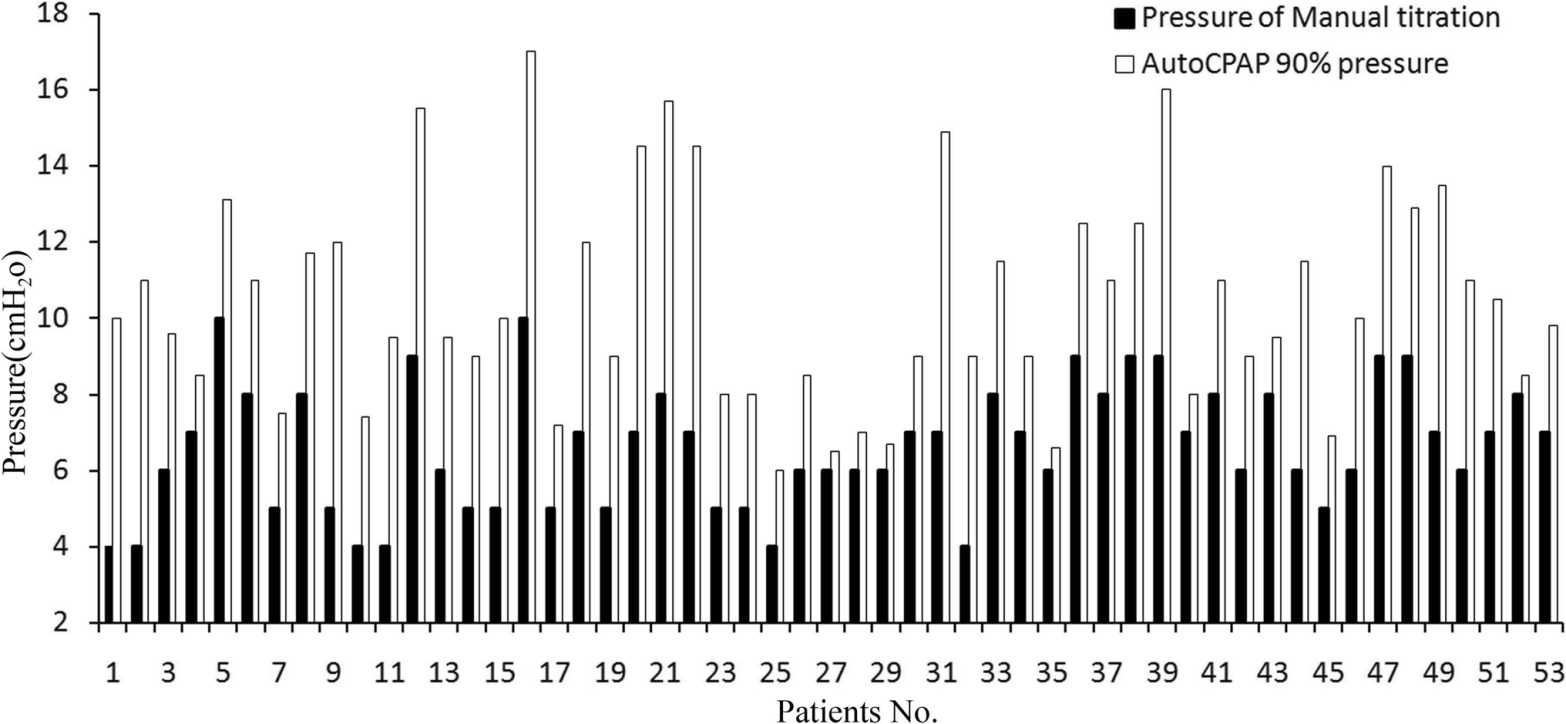
The pressure from manual titration and automatic titration

Both manual titration and automatic titration of CPAP pressure were performed in 53 patients with OSA (study 2). The mean CPAP pressure derived from manual titration (6.6 ± 1.7 cmH_2_O) was significantly lower than that derived from automatic titration (10.5 ± 2.8 cmH20, *p* < 0.001) (Fig. 2), as determined by the 90% of the pressure time.

### The effect of simple CPAP on patients with OSA

In total, simple CPAP was used in 105 patients and showed that it could satisfactorily improve sleep structure. The mean AHI and ODI normalized on treatment with simple CPAP; the AHI and ODI were 40.7 ± 2.3 and 28.4 ± 2.1events/h at baseline, respectively, and were 2.5 ± 0.3 and 1.7 ± 0.2 events/h while on simple CPAP, respectively (all *p* < 0.001). The arousal index decreased from 31.8 ± 2.0 to 11.8 ± 0.5 events/h on treatment (*p* < 0.001; Table 3 and online supplementary table E-3).

**Table 3:** Sleep structure and respiratory events before and after treatment with the simple CPAP device in 105 patients (mean ± SE)

Parameter	Baseline	Simple CPAP	*p* value
Total record time (min)	495.9 ± 4.8	468.6 ± 5.3	0.000
Total sleep time (min)	392.2 ± 6.4	382.5 ± 6.7	0.232
Sleep onset latency (min)	20.5 ± 2.3	22.4 ± 2.3	0.524
Sleep efficiency (%)	79.6 ± 1.3	81.7 ± 1.2	0.113
Arousal index (events/h)	31.8 ± 2.0	11.8 ± 0.5	0.000
REM sleep (%)	17.2 ± 0.7	21.8 ± 0.6	0.000
Stage 1 (%)	24.5 ± 1.4	13.7 ± 0.8	0.000
Stage 2 (%)	51.6 ± 1.3	51.5 ± 1.0	0.907
Stage 3 (%)	6.6 ± 0.7	13.0 ± 0.9	0.000
AHI (events/h)	40.7 ± 2.3	2.5 ± 0.3	0.000
OAI (events/h)	18.4 ± 1.7	0.6 ± 0.1	0.000
CAI (events/h)	2.4 ± 0.3	0.5 ± 0.1	0.000
MAI (events/h)	4.7 ± 0.7	0.1 ± 0.0	0.000
HI (events/h)	15.1 ± 0.9	1.4 ± 0.2	0.000
Sleep time mean SaO_2_ (%)	94.3 ± 0.3	96.3 ± 0.1	0.000
Lowest SaO_2_ (%)	76.9 ± 1.2	90.8 ± 0.4	0.000
ODI (events/h)	28.4 ± 2.1	1.7 ± 0.2	0.000

### Comparison between simple CPAP and conventional fixed CPAP

Fifty-two patients completed treatment both with conventional fixed CPAP and simple CPAP devices. The AHI, ODI, and arousal index improved significantly both with conventional fixed CPAP and simple CPAP. There were no significant differences between the treatment effects using simple CPAP and conventional fixed CPAP (AHI, 3.1 ± 0.6 events/h vs 3.3 ± 0.6 events/h; ODI 2.1 ± 0.4 events/h vs 2.3 ± 0.4 events/h; arousal index 12.2 ± 0.7 events/h vs 11.0 ± 0.6 events/h; lowest SaO_2_ 90.7% ± 0.7% vs 90.3% ± 0.6% respectively, all *p* > 0.05). Moreover, sleep structure including REM sleep and non-REM sleep stage 3 was also similar (Table 4 and online supplementary table E-4). Blinded to device allocation, 26.9% patients preferred the treatment with simple CPAP, and 36.5% patients preferred treatment with fixed pressure CPAP, whereas 36.6% of patients reported no preference (*p* = 0.218).

**Table 4:** Effects of simple CPAP and fixed CPAP in 52 patients (mean ± SE)

Parameter	Baseline	Simple CPAP	Conventional CPAP	*p* value^†^
Total record time (min)	490.2 ± 7.2	474.0 ± 4.9^a^	482.3 ± 7.0	0.300
Total sleep time (min)	400.2 ± 8.8	391.9 ± 8.2	398.1 ± 9.2	0.514
Sleep onset latency (min)	14.6 ± 1.8	22.5 ± 3.4^a^	20.8 ± 3.9	0.643
Sleep efficiency (%)	82.0 ± 1.6	82.7 ± 1.5	82.8 ± 1.8	0.944
Arousal index (events/h)	33.3 ± 2.8	12.2 ± 0.7^a^	11.0 ± 0.6^a^	0.080
REM sleep (%)	16.6 ± 0.9	21.1 ± 0.6^a^	21.7 ± 0.7^a^	0.479
Stage 1 (%)	28.7 ± 2.4	16.3 ± 1.3^a^	16.1 ± 1.1^a^	0.798
Stage 2 (%)	48.6 ± 2.0	51.2 ± 1.5	50.9 ± 1.5	0.755
Stage 3 (%)	6.0 ± 0.9	11.4 ± 1.2^a^	11.3 ± 1.2^a^	0.843
AHI (events/h)	41.6 ± 3.2	3.1 ± 0.6^a^	3.3 ± 0.6^a^	0.686
OAI (events/h)	19.2 ± 2.6	0.7 ± 0.2^a^	1.0 ± 0.4^a^	0.243
CAI (events/h)	2.6 ± 0.5	0.6 ± 0.9^a^	0.5 ± 0.1^a^	0.791
MAI (events/h)	3.4 ± 0.8	0.1 ± 0.0^a^	0.1 ± 0.0^a^	0.119
HI (events/h)	16.3 ± 1.4	1.8 ± 0.4^a^	1.6 ± 0.3^a^	0.504
Sleep time mean SaO_2_ (%)	94.2 ± 0.4	96.3 ± 0.2^a^	96.5 ± 0.2^a^	0.184
Lowest SaO_2_ (%)	77.2 ± 1.7	90.7 ± 0.7^a^	90.3 ± 0.6^a^	0.512
ODI (events/h)	29.1 ± 3.0	2.1 ± 0.4^a^	2.3 ± 0.4^a^	0.521

^†^*p* value indicates the difference between simple CPAP and conventional fixed CPAP; ^a^*p* < 0.05 compared with baseline

### Comparison between simple CPAP and autoCPAP

Fifty-three patients with OSA were treated using both simple CPAP and autoCPAP devices. The AHI, ODI, and arousal index improved significantly both with simple CPAP and autoCPAP. There was no difference between treatment with simple CPAP or autoCPAP in AHI (1.9 ± 0.2 events/h vs 1.9 ± 0.2 events/h) and ODI (1.3 ± 0.2 vs 1.3 ± 0.2 times/h, *p* > 0.05). The arousal index and lowest SaO_2_ during treatment with the two devices were also similar (11.0 ± 0.7 events/h vs 9.9 ± 0.7 events/h for arousal index and 90.9% ± 0.5% vs 91.2% ± 0.5% for lowest SaO_2_, *p* > 0.05; Table 5 and online supplementary table E-5). The proportion of patients who preferred treatment with the simple CPAP (38%) was similar to that who preferred treatment with autoCPAP (34%), and 28% of the patients reported no preference (*p* = 0.646).

**Table 5:** Effects of simple CPAP and autoCPAP in 53 patients (mean ± SE)

Parameter	Baseline	Simple CPAP	autoCPAP	*p* value^†^
Total record time (min)	501.6 ± 6.4	463.3 ± 9.3^a^	482.0 ± 7.6^a^	0.088
Total sleep time (min)	384.3 ± 9.2	373.3 ± 10.5	390.8 ± 7.8	0.181
Sleep onset latency (min)	26.3 ± 4.1	22.3 ± 3.1	22.9 ± 5.1	0.929
Sleep efficiency (%)	77.2 ± 2.0	80.8 ± 1.8	81.6 ± 1.5^a^	0.696
Arousal index (events/h)	30.2 ± 2.7	11.0 ± 0.7^a^	9.9 ± 0.7^a^	0.054
REM sleep (%)	17.8 ± 1.0	22.6 ± 0.9^a^	24.5 ± 1.0^a^	0.059
Stage 1 (%)	20.5 ± 1.3	11.2 ± 0.8^a^	12.0 ± 0.9^a^	0.312
Stage 2 (%)	54.6 ± 1.6	51.7 ± 1.3^a^	50.3 ± 1.3^a^	0.252
Stage 3 (%)	7.2 ± 1.0	14.6 ± 1.2^a^	12.8 ± 1.4^a^	0.061
AHI (events/h)	39.8 ± 3.4	1.9 ± 0.2^a^	1.9 ± 0.2^a^	0.915
OAI (events/h)	17.6 ± 2.3	0.5 ± 0.1^a^	0.3 ± 0.1^a^	0.19
CAI (events/h)	2.2 ± 0.4	0.4 ± 0.1^a^	0.4 ± 0.1^a^	0.585
MAI (events/h)	6.0 ± 1.2	0.1 ± 0.0^a^	0.1 ± 0.0^a^	0.87
HI (events/h)	13.9 ± 1.2	1.0 ± 0.1^a^	1.1 ± 0.1^a^	0.438
Sleep time mean SaO_2_ (%)	94.4 ± 0.3	96.2 ± 0.2^a^	96.3 ± 0.2^a^	0.403
Lowest SaO_2_ (%)	76.5 ± 1.7	90.9 ± 0.5^a^	91.2 ± 0.5^a^	0.561
ODI (events/h)	27.7 ± 3.0	1.3 ± 0.2^a^	1.3 ± 0.2^a^	0.953

^†^*p* value indicates the difference between simple CPAP and autoCPAP; ^a^*p* < 0.05 compared with baseline

## Discussion

Our results show that the therapeutic effect using a simple CPAP device is non-inferior to either conventional fixed pressure CPAP or autoCPAP for the treatment of patients with OSA, judged by the sleep architecture, conventional indices of sleep-disordered breathing, and sleep fragmentation. Blinded to the device allocation, patients expressed no specific preferences for any of the devices used in the study.

### Clinical significance of the findings

Given that our study and patient preference failed to show an advantage for state-of-the-art CPAP devices, it is worthwhile to consider the differences in the functional components of these devices. One function is pressure release which provides a temporary drop in airway pressure during early expiration. Because normal expiration is passive and relied on elastic recoil of the lung which is largest at early expiration and becomes smaller over the expiration, an ideal pressure release should be arranged at the later rather than the early part of expiration for facilitating expiration during CPAP. However, pressure release is usually developed at the early expiration for the modern CPAP. It is not surprising that CPAP with pressure release function may not improve expiration during CPAP. Indeed, we and others have previously shown that the efficacy of CPAP treatment and preferences for CPAP devices with or without pressure release is similar [18, 19].

Similarly, since airway closure is more likely when supine or in REM sleep, it could be hypothesized that at these times, therapeutic pressures need to be higher to maintain airway patency. This was the origin of the concept of autoCPAP which is able to track the resistance and adjust the pressures required to maintain airway patency and, thus, reduce mean pressures for parts of the night during CPAP treatment [20]. However, pressure delivered by autoCPAP depends on sensitivity and accuracy in detecting changes in airway resistance, which may be influenced by many factors including flow-derived algorithm used for automatic titration and frequency of arousals [21]. Indeed, it is shown that pressure titrated by autoCPAP is usually 2–3 cmH_2_O higher rather than lower than that obtained by manual titration, although the treatment efficacy and preference for the pressure derived from both titrations are similar [14]. Other study also showed that pressure derived from 95th percentile of treatment pressure during APAP therapy was usually 2 cmH_2_O higher than that from manual titration [22]. Consequently, it is not surprising that many studies show that the percentage of patients who prefer to be treated by fixed CPAP is actually more than or similar to those treated by autoCPAP [23–25].

The third function present in most current CPAP devices, which was deliberately omitted from our simple device to reduce cost, was a pressure ramp. Theoretically, if a CPAP device could respond automatically to sleep onset and increase the airway pressure to the required level to maintain upper airway patency, the ramp function might be useful to improve comfort prior to sleep onset. However, this is hypothetical as current commercially available CPAP devices do not have the ability to detect sleep onset, and the times set for pressure ramps are necessarily arbitrary, and this may be why we found no difference in sleep latency and overall quality or patient preference between the devices with and without a pressure ramp. Consistent with our observation, there are few data to support that pressure ramps improve patients’ long-term compliance to CPAP [26].

The humidification function for CPAP devices may be useful for some patients, particularly those who are under treatment with full-face mask rather than nasal mask and breathe through their mouth at night. However, CPAP increases lung volume, but it does not actually increase ventilation through the nose and it does not necessarily generate airway dryness when breathing room air. Indeed, many studies showed that humidification did not significantly improve long-term compliance [27, 28].

CPAP devices with fixed pressures may not be adjusted to increase pressure in the future, perhaps in response to weight gain. However, with good routine multidisciplinary clinical advice and management, this may not be a significant problem. Effective treatment of OSA should go beyond CPAP to encompass dietetic advice and weight loss in patients who are overweight [29]. It has been reported that weight in patients with OSA changes little during CPAP treatment [30, 31], and in long-term clinical trials with careful follow-up, CPAP pressures require little change following initiation of the treatment [7].

Although we did not record long-term compliance in a large population, it has previously been shown that initial tolerance to CPAP treatment predicts long-term compliance of CPAP treatment [32, 33]. In the current study, patients had similar preferences for autoCPAP, fixed pressure CPAP, and simple CPAP suggesting that long-term compliance to simple CPAP could be similar to autoCPAP or fixed pressure CPAP, although a further study is required to determine the long-term compliance of the simple CPAP.

## Conclusion

In summary, we found that the use of a simple CPAP device was not different to “state-of-the-art” CPAP devices for treating indices of sleep architecture in patients with OSA who required pressures up to 10cmH_2_O.

### Supplementary Information

Below is the link to the electronic supplementary material.

Supplementary file1 (ZIP 1292 KB)

## Data Availability

The datasets used and/or analyzed during the current study are available from the corresponding author on reasonable request.
