# Supplementary material for: Treatment of obstructive sleep apnea with a simple CPAP device
Source: Sleep Breath. 2023 May 22;27(6):2351–9. doi: 10.1007/s11325-023-02823-2 (PMC10656318; doi:10.1007/s11325-023-02823-2)
Supplement: Supplementary file 1 — Supplementary file1 (ZIP 1292 KB) [file 11325_2023_2823_MOESM1_ESM.zip › table E-3.pdf]

NO.	Total record time (min)		Total sleep time (min)		Sleep onset latency (min)		Sleep Efficiency (%)		Arousal index (events/h)		REM sleep (%)		Stage 1 (%)		Stage 2 (%)		Stage 3 (%)		AHI (events/h)		OAI（(events/h)		CAI（(events/h)		MAI (events/h)		HI (events/h)		Wake time Mean SaO2 (%)		Sleep time Mean SaO2 (%)		Lowest SaO2 (%)		ODI (events/h)	
	Baseline	simple CPAP	Baseline	simple CPAP	Baseline	simple CPAP	Baseline	simple CPAP	Baseline	simple CPAP	Baseline	simple CPAP	Baseline	simple CPAP	Baseline	simple CPAP	Baseline	simple CPAP	Baseline	simple CPAP	Baseline	simple CPAP	Baseline	simple CPAP	Baseline	simple CPAP	Baseline	simple CPAP	Baseline	simple CPAP	Baseline	simple CPAP	Baseline	simple CPAP	Baseline	simple CPAP
1	469.0	409.0	372.5	341.0	0.0	4.0	79.4	83.4	26.6	10.0	16.5	16.6	9.4	13.3	66.4	60.6	0.0	9.5	17.6	0.5	4.5	0.0	0.0	0.2	0.0	0.0	13.0	0.4	97	97	96	97	92	94	3.3	0.3
2	546.0	508.0	329.0	317.5	8.5	36.5	60.3	62.5	20.5	16.3	6.4	23.1	58.8	23.9	34.8	47.1	0.0	9.3	50.9	3.8	37.8	0.0	0.0	0.2	0.0	0.0	13.1	3.6	96	97	95	96	83	89	24.1	1.8
3	474.0	446.0	416.0	377.5	10.5	13.0	87.8	84.6	8.7	13.4	23.4	19.9	42.8	14.2	31.7	64.5	2.0	1.5	17.9	1.0	0.9	0.0	1.0	0.2	0.0	0.0	16.0	0.8	94	96	93	95	82	92	21.0	1.3
4	532.0	479.0	502.0	453.0	12.0	9.0	94.4	94.6	18.2	12.5	1.6	18.3	27.7	18.9	61.2	45.0	9.6	17.8	16.4	0.1	2.7	0.0	0.2	0.0	0.2	0.0	15.9	0.1	96	96	95	95	90	93	5.3	0.5
5	417.0	431.0	290.5	393.5	15.0	0.0	69.7	91.3	24.0	5.9	17.9	28.0	20.1	9.9	52.0	49.8	10.0	12.3	28.9	2.3	6.0	0.0	2.9	1.2	0.6	0.5	19.4	0.6	97	98	96	98	74	95	18.0	0.8
6	489.0	513.0	439.5	483.0	4.5	7.0	89.9	94.2	27.7	8.6	17.3	21.8	26.1	11.3	56.7	65.3	0.0	1.6	52.2	3.0	19.9	0.7	1.5	1.2	3.1	0.0	41.7	1.0	95	96	95	96	83	91	44.8	1.3
7	490.0	491.0	374.5	382.0	19.0	28.5	76.4	77.8	18.3	9.9	12.3	22.9	19.5	15.2	64.2	57.9	4.0	4.1	22.8	2.4	10.9	0.2	0.8	0.8	0.6	0.0	10.4	1.4	93	96	93	95	80	90	16.0	2.0
8	411.0	468.0	357.0	413.5	11.5	9.0	86.9	88.4	11.4	16.1	29.1	24.3	14.4	10.3	47.5	57.4	9.0	8.0	9.9	1.5	0.0	0.0	1.3	1.2	0.0	0.0	8.2	0.3	99	97	98	97	94	95	2.4	1.5
9	455.0	432.0	439.5	375.5	9.0	31.0	96.6	86.9	43.8	31.2	18.1	20.4	12.3	13.3	64.8	64.6	4.8	1.7	45.9	26.0	28.7	6.2	1.2	0.8	11.9	0.6	4.1	18.4	96	97	92	97	68	86	48.9	12.8
10	503.0	487.0	402.5	391.5	26.5	6.5	80.0	80.4	12.2	9.0	32.8	18.0	33.9	22.2	33.2	59.6	0.1	0.1	12.4	0.8	1.9	0.3	1.5	0.2	0.0	0.0	8.2	0.3	96	97	96	96	87	95	5.3	0.6
11	450.0	432.0	426.5	382.5	7.5	6.0	94.8	88.5	7.7	5..2	15.4	20.1	15.5	6.9	68.9	72.9	0.2	0.0	15.6	0.3	4.2	0.0	3.1	0.0	1.0	0.0	7.3	0.3	93	98	93	97	80	95	13.2	0.0
12	537.0	450.0	469.5	360.0	32.5	7.5	87.4	80.0	65.3	16.0	11.8	17.8	40.0	14.2	48.0	60.4	0.1	7.6	84.1	7.2	40.0	0.0	7.8	5.5	25.6	0.2	11.8	1.5	94	96	91	95	66	80	73.8	5.5
13	487.0	493.0	434.5	466.5	20.5	3.0	89.2	94.6	16.3	11.2	21.5	16.4	12.8	4.6	49.7	54.9	16.0	24.1	16.2	0.8	0.6	0.0	1.4	0.6	0.0	0.0	14.2	0.1	96	99	95	98	87	95	7.2	0.6
14	439.0	519.0	409.0	379.5	7.5	73.5	93.2	73.1	67.2	9.0	14.5	29.1	53.7	12.4	31.8	53.1	0.0	5.4	78.3	6.3	75.0	1.1	0.4	0.3	0.6	0.3	2.2	4.6	88	97	82	96	47	89	75.0	4.1
15	552.0	467.0	430.5	414.0	8.0	20.5	78.0	88.7	59.5	15.5	8.2	25.5	65.5	31.9	26.2	39.0	0.0	3.6	68.0	4.5	15.1	0.6	16.3	0.1	4.9	0.0	31.8	3.8	96	96	95	96	76	93	59.6	3.4
16	522.0	492.0	278.5	440.5	33.0	15.0	55.7	89.5	80.4	10.6	6.6	24.6	50.4	18.8	40.6	44.0	2.3	12.5	75.8	2.5	0.0	0.5	0.4	1.1	0.0	0.4	48.8	0.4	97	98	96	98	83	94	22.0	1.3
17	624.0	455.0	406.5	273.5	38.5	22.5	65.1	60.1	31.3	7.5	15.9	19.4	18.2	10.8	55.4	58.7	10.6	11.2	33.1	0.4	18.0	0.0	0.1	0.0	0.0	0.0	14.9	0.4	95	97	94	97	83	94	19.4	0.1
18	492.0	457.0	405.5	374.0	3.0	19.0	82.4	81.8	42.9	6.7	10.0	23.4	35.6	10.6	47.3	53.5	7.0	12.6	63.0	4.2	24.4	0.0	12.3	0.5	4.3	0.0	22.0	3.7	96	96	95	94	85	87	51.5	4.1
19	481.0	488.0	423.0	457.5	1.0	9.0	87.9	93.8	21.6	20.1	14.5	20.9	24.3	16.0	61.1	61.5	0.0	1.6	19.7	3.1	2.1	0.0	0.0	0.0	0.0	0.0	17.6	3.1	95	96	94	95	79	75	17.1	3.7
20	567.0	515.0	545.0	454.0	4.5	34.0	96.1	88.2	9.6	4.6	16.0	25.2	9.0	6.7	48.1	45.9	27.0	22.1	10.5	0.1	1.8	0.0	0.3	0.1	0.0	0.0	10.8	0.0	97	98	96	98	85	98	7.7	0.2
21	551.0	577.0	487.5	432.0	23.5	135.5	88.5	74.9	55.5	14.3	11.4	19.3	55.5	14.2	33.1	62.6	0.0	3.8	70.2	4.0	32.4	0.8	3.9	1.0	4.2	0.0	26.3	2.2	96	98	96	97	90	92	32.5	2.4
22	457.0	463.0	393.5	383.5	33.0	45.0	86.1	82.8	49.3	9.5	13.9	25.8	34.9	15.5	34.9	40.8	16.3	17.9	56.1	0.9	39.8	0.3	0.2	0.0	1.1	0.2	12.2	0.5	97	99	95	98	60	95	43.4	0.6
23	543.0	456.0	460.0	388.5	10.5	7.0	84.7	85.2	7.7	15.8	18.7	21.2	7.9	28.1	48.5	28.3	24.9	22.4	10.3	3.4	3.3	1.5	0.0	0.0	0.0	0.0	7.0	1.9	97	98	96	97	80	86	7.4	2.8
24	545.0	456.0	346.0	369.5	4.5	11.5	63.5	81.0	33.5	15.4	16.6	21.4	26.2	18.9	44.8	34.5	12.4	25.2	33.6	0.8	0.2	0.0	0.0	0.0	0.0	0.0	25.2	0.8	93	93	92	93	85	88	17.2	0.9
25	478.0	510.0	354.5	312.0	24.5	29.0	74.2	61.2	32.0	11.0	11.8	16.2	11.0	13.6	69.8	50.2	7.3	20.0	36.1	1.5	13.4	0.0	0.0	0.0	0.2	0.0	22.5	1.5	94	96	93	95	74	92	32.8	1.0
26	492.0	418.0	438.5	252.5	6.5	27.0	89.1	60.4	81.8	15.9	11.1	17.6	7.6	10.1	77.8	41.6	3.5	30.7	91.9	7.6	55.3	0.0	3.4	1.2	8.5	0.5	30.6	5.9	91	97	89	96	40	80	83.8	5.3
27	522.0	478.0	416.5	377.0	13.0	29.0	79.8	78.9	48.8	12.6	13.2	18.4	56.8	30.8	24.4	40.1	5.6	10.7	57.2	4.5	20.0	0.6	6.6	0.0	10.1	0.0	20.5	3.8	98	95	90	94	66	88	52.6	4.9
28	502.0	486.0	345.5	342.0	9.5	33.5	68.8	70.4	16.8	17.9	20.3	17.7	20.8	10.5	52.7	56.1	6.2	15.6	26.0	0.7	0.2	0.0	0.2	0.4	0.0	0.2	21.3	0.2	94	96	94	96	81	93	15.8	0.1
29	465.0	453.0	260.0	368.0	21.0	10.5	55.9	81.2	10.2	6.5	12.1	18.6	19.4	20.1	67.9	59.6	0.6	1.6	35.3	3.3	5.8	1.6	3.2	0.2	0.2	0.0	26.1	1.5	95	97	95	96	81	93	18.9	1.1
30	394.0	482.0	369.5	394.0	3.5	17.5	93.8	81.7	33.6	16.9	24.4	24.9	19.5	7.5	45.3	42.5	10.8	25.1	41.1	2.7	30.4	1.4	0.8	0.2	2.6	0.0	10.4	1.2	96	97	96	97	60	91	28.7	2.1
31	537.0	415.0	440.0	385.0	9.5	11.0	81.9	92.8	21.1	11.8	35.5	26.1	19.5	12.2	36.4	43.0	8.6	18.7	31.0	2.5	17.5	0.8	0.7	0.3	1.6	0.2	11.2	1.2	96	98	96	97	73	93	22.1	2.4
32	535.0	520.0	489.0	481.0	0.0	30.0	91.4	92.5	85.6	16.6	20.7	28.2	31.9	4.9	47.4	48.9	0.0	18.1	91.4	8.0	66.1	0.6	5.0	2.4	18.4	0.6	2.0	4.4	95	96	92	96	70	88	87.1	6.7
33	357.0	408.0	276.5	277.5	18.0	26.5	77.5	68.0	20.6	8.9	19.0	21.3	26.6	18.2	52.1	56.9	2.4	3.6	34.7	0.0	9.8	0.0	1.7	0.0	0.9	0.0	22.4	0.0	96	97	94	97	81	94	22.0	0.0
34	424.0	507.0	379.5	408.0	0.0	11.5	89.5	80.5	19.6	12.5	27.4	21.9	14.1	16.2	54.8	59.8	3.7	2.1	15.5	0.9	3.5	0.0	0.2	0.1	0.2	0.0	16.1	0.7	96	98	96	97	87	93	8.6	0.5
35	474.0	502.0	402.5	331.5	8.5	3.5	84.9	66.0	46.8	17.9	16.0	14.9	19.8	10.6	64.2	72.1	0.0	2.4	48.3	2.4	23.4	1.1	0.7	0.0	1.0	0.2	23.1	1.1	98	98	98	97	87	92	26.5	1.9
36	504.0	445.0	426.0	322.0	17.0	104.0	84.5	72.4	27.2	10.8	12.0	22.0	31.0	27.5	52.3	43.8	4.7	6.7	35.1	1.9	7.2	0.6	5.8	0.6	2.1	0.0	21.2	0.7	93	97	92	96	77	95	21.8	0.3
37	480.0	503.0	381.0	341.0	16.0	9.0	79.4	67.8	27.6	12.7	11.5	19.8	46.3	36.5	41.3	41.3	0.8	2.3	38.7	1.1	17.3	0.7	2.4	0.4	4.3	0.0	14.8	0.0	97	98	97	98	81	96	25.0	0.1
38	560.0	483.0	338.5	414.5	12.5	31.0	60.4	85.8	38.5	21.9	26.4	17.4	19.2	18.6	36.6	48.5	17.7	15.6	46.6	15.2	40.9	4.2	0.4	1.6	0.0	0.3	4.7	9.1	96	97	92	95	73	79	46.1	12.9
39	561.0	471.0	354.0	284.5	24.0	13.0	63.1	60.4	26.1	8.6	9.2	8.6	40.8	35.3	46.5	38.8	3.5	17.2	45.4	1.5	16.4	0.2	5.3	0.6	4.9	0.2	18.8	0.4	95	98	94	98	68	91	27.9	0.4
40	455.0	482.0	436.5	465.0	5.5	8.0	95.9	96.5	50.6	9.0	14.2	17.6	15.5	9.2	66.9	62.2	3.4	11.0	59.2	1.4	29.4	0.1	14.0	0.5	10.4	0.0	6.6	0.8	97	98	95	97	74	88	54.5	1.4
41	510.0	548.0	468.0	528.0	6.5	4.0	91.8	96.4	47.6	23.1	9.7	23.1	26.0	17.0	62.1	51.0	2.2	8.8	49.5	3.8	6.2	0.6	3.1	0.6	1.4	0.5	38.8	2.2	96	96	94	94	76	84	37.4	3.9
42	444.0	510.0	271.5	449.5	10.5	9.0	61.1	88.1	54.1	8.4	7.9	13.3	82.1	31.4	9.9	55.3	0.0	0.0	79.6	1.3	53.3	0.3	8.4	0.1	14.6	0.0	1.9	0.9	95	97	94	96	68	83	47.7	0.8
43	484.0	487.0	465.0	404.5	4.5	46.5	96.1	83.1	63.2	9.6	16.8	21.0	22.3	7.9	61.0	54.6	0.0	16.4	66.6	11.6	39.5	4.9	1.4	3.0	24.0	1.0	1.7	2.7	96	97	87	96	34	87	61.4	6.8
44	441.0	424.0	408.5	416.0	4.5	3.5	92.6	98.1	12.5	15.6	20.6	22.5	15.9	17.8	56.7	43.8	6.9	16.0	10.7	0.1	0.9	0.1	0.0	0.0	0.3	0.0	8.4	0.0	98	98	97	98	80	95	7.2	0.1

45	484.0	470.0	403.5	380.5	33.5	38.5	83.4	81.0	18.3	8.5	16.7	13.5	40.4	38.9	36.4	43.0	6.4	4.6	16.7	0.5	2.4	0.0	2.5	0.5	0.4	0.0	11.3	0.0	97	97	97	97	80	95	10.7	0.6
46	515.0	460.0	432.0	331.0	65.5	39.0	83.9	72.0	26.9	16.1	17.8	13.7	21.6	6.3	50.2	60.7	10.3	19.2	26.5	0.5	3.8	0.0	0.6	0.5	0.1	0.0	23.6	0.0	98	96	96	96	87	95	16.3	0.1
47	452.0	442.0	381.5	422.5	14.5	11.0	84.4	95.6	51.9	6.2	19.9	30.8	20.1	3.8	44.6	40.2	15.5	25.2	62.8	1.3	43.4	0.0	1.7	0.9	3.0	0.0	14.6	0.4	97	98	93	98	67	92	51.4	1.4
48	507.0	519.0	425.0	491.5	44.5	17.5	83.8	94.7	20.6	6.6	23.5	23.0	46.8	41.0	28.5	24.5	1.2	11.5	57.7	6.6	56.9	6.1	0.0	0.1	0.3	0.0	0.6	0.4	98	98	98	99	86	87	12.5	1.5
49	477.0	482.0	455.0	456.0	9.0	15.5	95.4	94.6	8.2	4.1	11.9	28.6	12.1	10.4	64.5	38.6	11.5	22.4	19.4	0.0	8.8	0.0	0.0	0.0	0.3	0.0	10.3	0.0	95	96	94	95	83	95	17.7	0.0
50	486.0	457.0	421.5	438.5	26.0	10.0	86.7	96.0	27.3	13.1	20.0	28.4	26.7	15.7	48.5	43.9	4.7	12.0	35.3	2.3	17.2	0.1	0.4	0.1	1.1	0.0	15.3	2.1	94	96	93	95	82	90	26.4	2.1
51	370.0	463.0	273.5	392.5	4.5	16.5	73.9	84.8	24.2	10.5	27.2	27.4	6.4	7.1	50.5	65.2	15.9	0.3	30.9	0.3	16.0	0.2	0.4	0.2	0.0	0.0	14.5	0.0	96	97	96	97	91	94	4.7	0.1
52	545.0	439.0	460.0	410.0	4.5	10.5	84.4	93.4	31.6	6.0	14.6	17.1	56.1	8.3	27.8	56.5	1.5	18.2	62.9	0.3	24.1	0.0	11.5	0.0	8.2	0.1	32.0	0.1	95	95	95	94	88	91	9.0	0.0
63	514.0	515.0	374.0	346.0	19.5	12.5	72.8	67.2	12.4	14.7	14.8	14.7	31.0	21.2	46.9	43.6	7.2	20.4	20.9	1.4	1.3	0.0	2.4	0.5	0.2	0.0	17.0	0.9	98	98	97	98	81	92	9.9	0.3
64	518.0	451.0	399.5	380.0	30.5	0.0	77.1	84.3	13.8	11.5	16.0	17.6	10.0	8.2	55.2	49.9	18.8	24.3	16.7	0.9	8.9	0.3	0.0	0.0	0.0	0.0	8.3	0.6	96	98	95	97	83	94	8.2	0.7
65	464.0	360.0	377.5	337.0	1.0	0.0	81.4	93.6	27.4	14.4	12.8	16.3	33.0	22.1	36.0	34.6	18.1	27.0	38.8	9.4	8.3	4.1	6.8	1.2	7.5	1.4	16.2	2.7	95	95	95	95	85	86	26.4	8.0
66	464.0	500.0	422.0	370.0	17.5	47.5	90.9	74.0	26.8	10.6	24.3	24.1	20.9	12.3	48.6	55.4	6.3	8.2	26.7	7.0	10.8	0.0	3.0	2.4	6.7	0.0	7.0	4.5	94	95	94	95	74	89	18.6	3.6
67	510.0	440.0	253.5	303.5	39.0	24.0	49.7	69.0	32.5	5.3	2.0	4.4	49.9	13.3	48.1	67.4	0.0	14.8	64.5	0.2	21.1	0.2	4.5	0.0	18.0	0.0	20.8	0.0	96	97	96	97	84	95	23.2	0.0
68	530.0	449.0	433.5	358.5	31.5	48.0	81.8	79.8	24.9	5.6	20.8	31.1	13.3	4.0	65.9	60.1	0.1	4.7	30.4	0.3	9.6	0.0	7.1	0.3	4.6	0.0	15.9	0.0	97	94	97	97	82	96	23.3	0.0
69	523.0	486.0	375.0	402.0	26.5	21.0	71.7	82.7	25.0	21.0	22.1	21.0	27.1	18.7	50.8	51.0	0.0	9.3	37.1	2.1	27.7	0.6	4.2	0.7	3.7	0.0	1.6	0.7	94	95	94	95	86	88	13.1	0.1
71	388.0	511.0	358.0	447.0	1.0	19.5	92.3	87.5	45.6	6.8	18.6	22.7	31.0	6.2	50.4	56.4	0.0	14.8	57	0.8	32.2	0.1	1.3	0.5	17.6	0.1	5.9	0.0	94	96	93	96	75	92	53.9	0.5
72	488.0	444.0	399.5	305.5	18.0	0.0	81.9	68.8	28.1	18.0	2.8	11.6	19.5	20.6	75.3	61.7	2.4	6.1	25.2	3.5	8.1	2.0	0.2	0.0	0.0	0.0	17.0	1.6	94	92	94	93	85	92	18.8	1.9
73	530.0	518.0	346.5	471.0	141.5	14.0	65.4	90.9	37.2	14.4	20.5	27.1	21.2	3.5	45.0	47.3	13.3	22.1	33.1	1.0	9.5	0.1	0.9	0.3	2.1	0.1	17.9	0.5	96	96	95	95	86	90	17.6	1.0
74	517.0	366.0	393.5	275.0	28.5	12.5	76.1	75.1	13.4	4.2	20.5	27.3	27.7	11.1	41.3	57.6	10.5	4.0	18.8	0.2	6.3	0.0	1.1	0.2	1.7	0.0	9.8	0.0	97	98	96	98	85	96	10.3	0.6
75	493.0	440.0	430.5	417.0	18.5	8.5	87.3	94.8	77.6	22.7	17.3	22.2	27.5	10.4	54.6	48.9	0.6	18.5	91.8	4.3	67.3	2.2	2.5	0.7	15.1	0.0	7.6	1.4	97	96	94	97	63	91	66.8	1.9
76	454.0	519.0	243.0	342.5	11.0	29.0	53.5	66.0	28.0	11.4	8.6	18.7	20.2	9.9	38.9	47.4	32.3	23.9	44.4	4.0	0.2	0.2	1.7	0.0	0.0	0.0	42.5	3.9	96	98	96	97	87	83	22.1	2.4
77	485.0	449.0	418.5	410.5	5.5	7.5	86.3	91.4	31.4	15.9	8.6	24.1	30.0	20.5	57.0	36.3	4.4	19.1	31.4	0.0	16.8	0.0	4.9	0.0	5.0	0.0	8.1	0.0	96	98	96	97	91	94	14.1	0.0
78	450.0	444.0	392.5	384.5	32.5	19.5	87.2	86.6	36.2	15.5	7.6	22.5	7.9	11.2	83.6	54.4	0.9	12.0	33.2	1.9	17.7	0.3	0.2	0.2	0.8	0.0	14.5	1.4	97	95	96	95	86	90	22.3	1.0
79	422.0	523.0	398.0	501.5	1.0	9.5	94.3	95.9	65.1	11.1	27.6	28.4	14.4	16.0	57.9	54.0	0.0	1.6	72.1	3.1	36.2	1.6	2.0	0.2	28.5	0.4	5.5	1.0	95	96	90	96	51	91	66.4	1.2
80	498.0	533.0	257.0	394.5	24.5	77.0	51.6	74.0	13.6	18.8	11.9	17.0	37.4	17.2	43.8	58.0	7.0	7.7	19.8	3.5	6.8	1.2	0.0	0.9	0.0	0.2	13.1	1.2	92	94	92	93	80	88	8.7	2.9
81	505.0	406.0	432.0	351.0	22.5	40.0	85.5	86.5	54.1	6.2	20.7	27.4	32.2	10.4	46.2	53.4	0.9	8.8	70	1.7	31.8	0.0	6.4	1.2	15.4	0.0	27.5	0.5	94	96	90	95	63	91	60.7	2.7
82	459.0	378.0	425.0	365.0	11.0	4.0	92.6	96.6	24.9	6.2	24.1	22.2	10.2	4.9	41.9	32.7	23.8	40.1	30.9	1.5	7.6	0.0	0.1	0.0	0.7	0.0	22.4	1.5	97	98	97	97	76	90	22.0	1.1
83	577.0	456.0	436.5	369.5	115.5	18.5	75.6	81.0	72.6	12.9	10.7	34.0	16.2	3.5	73.2	41.1	0.0	21.4	78.1	2.4	50.4	0.5	2.5	0.8	24.7	0.0	0.4	1.1	94	97	90	96	58	89	65.2	1.6
84	444.0	461.0	389.0	410.5	6.0	14.0	87.6	89.0	24.5	5.6	11.8	24.2	24.2	11.4	53.7	53.5	10.3	10.8	32.9	1.3	17.7	0.1	0.0	0.6	0.0	0.0	15.1	0.6	94	97	93	96	73	91	25.0	0.7
85	476.0	383.0	452.0	348.5	2.5	0.0	95.0	91.0	35.0	6.3	22.3	27.8	16.2	8.2	57.1	40.6	4.4	23.4	47.5	1.4	39.4	0.0	2.0	0.7	4.9	0.2	1.4	0.5	94	97	93	96	54	90	43.1	0.9
87	413.0	588.0	392.5	412.0	9.0	14.5	95.0	70.1	9.2	8.1	17.7	15.7	9.0	10.8	55.5	72.8	17.7	0.7	6.1	0.6	1.1	0.0	0.6	0.1	0.0	0.1	4.4	0.3	94	96	94	95	77	92	6.0	0.4
88	515.0	469.0	446.0	352.5	9.0	17.0	86.6	75.2	22.4	10.4	17.9	22.1	7.5	10.5	71.6	65.7	2.9	1.7	44	2.0	26.5	0.7	1.1	0.0	3.6	0.0	14.5	1.4	96	97	96	97	64	91	32.8	0.1
89	508.0	410.0	484.0	351.5	9.5	46.0	95.3	85.7	7.9	19.6	22.4	10.2	7.2	5.0	58.8	60.9	11.6	23.9	6.6	0.7	0.1	0.0	0.4	0.3	0.0	0.0	6.1	0.3	96	97	96	96	85	93	2.8	2.1
91	620.0	457.0	499.0	431.5	33.0	3.5	80.5	94.4	15.4	5.0	17.8	18.1	11.3	10.1	68.6	51.9	2.2	19.9	26.7	1.3	0.7	0.0	0.0	0.0	0.0	0.0	26.8	1.3	95	96	94	96	82	90	19.0	0.9
92	434.0	476.0	394.5	412.0	8.0	22.5	90.9	86.6	9.3	10.8	28.4	26.7	12.2	8.5	44.5	47.1	15.0	17.7	18.7	2.3	7.5	0.6	0.8	1.7	0.5	0.0	10.0	0.0	98	98	98	98	88	92	8.7	0.3
93	535.0	447.0	359.0	349.5	23.5	28.0	67.1	78.2	16.5	8.8	15.3	24.9	18.1	4.1	66.4	59.7	0.1	11.3	29.2	1.0	7.0	0.0	0.8	0.2	0.5	0.0	19.0	0.9	95	95	95	95	77	89	15.8	1.4
96	558.0	375.0	367.5	209.0	8.5	33.0	65.9	55.7	14.4	12.4	14.3	10.0	16.3	12.0	66.7	64.4	2.7	13.6	16.2	1.1	0.0	0.0	1.3	0.0	0.0	0.0	14.9	1.1	95	97	95	96	88	92	7.6	0.3
99	480.0	539.0	397.5	379.5	25.0	27.0	82.8	70.4	17.0	13.6	19.5	21.1	29.2	17.1	42.1	58.0	9.2	3.8	14.79	1.9	0.2	1.1	0.0	0.0	0.0	0.0	15.8	0.8	95	93	94	93	89	91	5.4	0.0
100	419.0	471.0	397.5	459.0	2.0	5.0	94.9	97.5	32.2	15.1	17.2	26.1	25.8	4.9	41.4	43.8	15.6	25.2	41.66	1.2	24.5	0.3	2.7	0.1	0.6	0.0	13.9	0.8	96	97	95	96	60	92	37.0	2.8
101	536.0	464.0	392.5	274.5	0.0	24.5	73.2	59.2	8.0	5.3	25.6	21.5	9.0	14.6	49.0	35.5	16.3	28.4	10.7	0.2	0.9	0.0	0.2	0.0	0.0	0.0	9.5	0.2	97	95	96	95	87	93	5.6	0.0
102	534.0	416.0	295.5	341.0	28.0	6.0	55.3	82.0	10.0	7.9	20.1	23.6	20.6	22.3	47.0	44.6	12.2	9.5	24	0.4	1.0	0.2	1.2	0.0	0.0	0.0	21.7	0.2	96	96	95	95	83	93	17.0	0.6
103	527.0	472.0	290.0	407.5	31.0	38.5	55.0	86.3	11.3	9.8	17.4	22.3	9.7	6.0	61.6	62.9	11.4	8.7	18.1	0.7	2.1	0.4	0.4	0.0	0.0	0.0	15.2	0.3	96	97	95	97	76	92	8.2	1.0
104	520.0	438.0	202.0	326.5	50.5	23.5	38.8	74.5	7.9	7.5	39.9	28.0	9.7	9.5	50.0	51.3	0.5	11.2	9.5	2.4	0.6	1.7	0.3	0.4	0.0	0.0	8.6	0.4	96	97	96	97	91	95	1.0	0.1
105	480.0	488.0	274.0	391.5	39.0	22.5	57.1	80.2	38.3	16.0	8.6	19.2	38.0	23.0	53.5	52.7	0.0	5.1	55.2	2.5	16.6	1.1	7.9	0.3	29.8	0.0	1.2	1.1	95	95	94	96	78	90	31.6	2.5
106	537.0	409.0	475.5	343.5	22.5	2.0	88.5	84.0	67.3	23.7	18.8	16.4	26.6	14.4	54.6	68.3	0.0	0.9	72.8	3.3	42.0	2.1	2.6	0.0	21.5	0.0	6.7	1.2	97	98	97	97	84	91	46.1	2.3
107	513.0	551.0	490.5	544.5	4.0	4.5	95.6	98.8	73.9	8.5	11.3	34.3	3.6	2.3	85.1	35.4	0.0	27.9	77.4	3.9	57.4	0.2	1.1	0.1	13.1	0.6	6.3	3.0	94	96	85	97	51	80	74.7	4.4
108	433.0	472.0	399.0	403.5	6.5	64.0	92.1	85.5	42.2	6.2	26.3	28.0	8.0	6.7	65.7	61.6	0.0	3.7	57.4	3.6	31.7	0.6	2.9	0.6	15.8	0.3	7.1	2.1	95	96	91	96	68	85	55.1	2.7
109	535.0	485.0	322.5	451.5	6.0	25.0	60.3	93.1	59.0	13.7	28.7	28.8	36.4	6.5	31.5	48.4	3.4	16.3	111.6	0.9	40.9	0.0	14.3	0.3	9.3	0.1	38.0	0.5	87	96	88	96	49	93	59.5	1.2

110	505.0	550.0	363.0	469.0	35.5	24.5	71.9	85.3	15.2	7.1	15.6	22.6	25.6	6.4	56.3	62.8	2.5	8.2	33.2	2.7	13.1	0.6	4.5	0.4	0.8	0.0	14.9	1.7	96	98	95	97	83	91	20.1	0.7
112	532.0	190.0	341.5	174.0	68.0	0.0	64.2	91.6	13.3	17.1	29.3	32.5	18.2	14.7	42.6	39.7	10.0	13.2	19.7	1.7	3.9	0.0	1.4	1.0	2.3	0.0	11.4	0.7	97	98	97	98	86	93	10.8	1.9
113	575.0	453.0	373.0	243.5	39.0	69.0	64.9	53.8	24.4	16.5	11.1	21.1	14.6	17.0	68.6	56.3	5.6	5.5	25.6	1.5	11.7	0.5	0.5	0.2	0.5	0.0	12.9	0.7	98	98	97	98	85	97	12.0	0.4
115	483.0	573.0	425.5	502.0	25.0	25.0	88.1	87.6	24.8	8.8	14.1	22.7	14.6	6.7	51.7	47.4	19.6	23.2	35.8	1.2	24.4	0.6	0.4	0.1	0.8	0.2	11.6	0.2	96	98	96	98	84	92	23.2	0.7
116	532.0	503.0	345.0	335.5	31.5	19.0	64.8	66.7	12.9	19.0	8.8	12.4	27.8	16.5	43.9	53.8	19.4	17.3	8.9	2.5	0.2	0.0	0.2	0.0	0.0	0.0	8.5	2.5	96	97	96	96	91	93	4.9	1.0
117	498.0	461.0	402.5	422.0	28.5	9.0	80.8	91.5	52.9	28.3	18.0	35.0	26.1	3.7	55.9	52.8	0.0	8.5	65.4	1.4	23.9	0.1	4.3	0.0	15.8	0.0	25.0	1.3	95	96	89	96	45	86	43.6	1.4
118	511.0	525.0	449.0	457.5	26.5	13.5	87.7	87.1	73.8	3.0	20.3	15.8	22.8	5.8	56.9	66.2	0.0	12.1	98.9	0.0	48.4	0.0	10.0	0.0	31.9	0.0	2.5	0.0	93	96	93	95	80	95	78.1	0.0
119	542.0	534.0	372.0	394.0	121.0	99.5	68.6	73.8	31.5	13.4	20.4	25.6	13.6	6.9	55.4	46.8	10.6	20.7	39.8	2.0	15.8	0.3	0.6	0.3	7.4	0.0	13.2	1.4	96	97	96	97	75	92	21.8	0.5
120	574.0	514.0	458.5	416.0	2.0	0.0	79.9	80.9	16.7	7.5	28.1	30.0	17.3	13.7	50.5	43.0	4.0	13.2	39	0.6	19.4	0.1	1.0	0.0	1.4	0.0	17.1	0.4	96	97	95	97	69	89	35.6	0.6
121	490.0	410.0	359.5	392.0	74.5	7.0	73.4	95.6	25.4	4.9	16.4	25.8	10.7	7.9	61.3	42.6	11.5	23.7	28	0.3	2.8	0.0	0.0	0.0	0.0	0.0	19.8	0.3	96	98	96	97	87	97	11.5	0.4
122	539.0	363.0	506.5	180.5	12.5	0.0	94.0	49.7	54.6	3.3	21.9	23.3	24.6	10.8	53.0	35.5	0.5	30.5	73	3.3	44.7	0.3	1.1	2.0	6.8	0.0	20.5	1.0	96	97	94	97	74	90	64.9	1.3
126	447.0	458.0	419.0	412.0	4.5	7.0	93.7	90.0	34.2	3.2	17.8	21.2	23.4	17.1	51.7	45.1	7.2	16.5	58.6	0.4	21.9	0.3	0.0	0.0	0.1	0.0	30.2	0.1	93	98	94	98	57	82	56.1	0.4
127	550.0	563.0	266.0	253.5	4.5	76.0	48.4	45.0	9.6	7.6	5.8	24.7	28.0	10.7	60.0	56.8	6.2	7.9	21.7	0.5	6.1	0.0	0.0	0.0	0.0	0.0	15.6	0.5	96	98	96	97	75	88	10.7	1.5
**Mean**	495.9	468.6	392.2	382.5	20.5	22.4	79.6	81.7	31.8	11.8	17.2	21.8	24.5	13.7	51.6	51.5	6.6	13.0	40.7	2.5	18.4	0.6	2.4	0.5	4.7	0.1	15.1	1.4	95.5	96.7	94.3	96.3	76.9	90.8	28.4	1.7
**SE**	4.8	5.3	6.4	6.7	2.3	2.3	1.3	1.2	2.0	0.5	0.7	0.6	1.4	0.8	1.3	1.0	0.7	0.9	2.3	0.3	1.7	0.1	0.3	0.1	0.7	0.0	0.9	0.2	0.2	0.1	0.3	0.1	1.2	0.4	2.1	0.2
