# Supplementary material for: Treatment of obstructive sleep apnea with a simple CPAP device
Source: Sleep Breath. 2023 May 22;27(6):2351–9. doi: 10.1007/s11325-023-02823-2 (PMC10656318; doi:10.1007/s11325-023-02823-2)
Supplement: Supplementary file 1 — Supplementary file1 (ZIP 1292 KB) [file 11325_2023_2823_MOESM1_ESM.zip › table E-2.pdf]

NO.	Total record time (min)	Total sleep time (min)	Sleep onset latency (min)	Sleep Efficiency (%)	Arousal index (events/h)	REM sleep (%)	Stage 1 (%)	Stage 2 (%)	Stage 3 (%)	AHI (events/h)	OAI(events/h)	CAI(events/h)	MAI (events/h)	HI (events/h)	Wake time Mean SaO2 (%)	Sleep time Mean SaO2 (%)	Lowest SaO2 (%)	3% ODI (events/h)
1	469.0	372.5	0.0	79.4	26.6	16.5	9.4	66.4	7.7	17.6	4.5	0.0	0.0	13.0	97	96	92	3.3
2	546.0	329.0	8.5	60.3	20.5	6.4	58.8	34.8	0.0	50.9	37.8	0.0	0.0	13.1	96	95	83	24.1
3	474.0	416.0	10.5	87.8	8.7	23.4	42.8	31.7	2.0	17.9	0.9	1.0	0.0	16.0	94	93	82	21.0
4	517.0	432.0	0.0	83.6	58.0	7.3	28.8	63.8	0.1	76.0	35.4	5.0	4.7	30.8	97	96	75	48.5
5	532.0	502.0	12.0	94.4	18.2	1.6	27.7	61.2	9.6	16.4	2.7	0.2	0.2	13.1	96	95	90	5.3
6	417.0	290.5	15.0	69.7	24.0	17.9	20.1	52.0	10.0	28.9	6.0	2.9	0.6	19.4	97	96	74	18.0
7	425.0	388.5	2.0	91.4	71.7	9.5	37.8	52.6	0.0	84.0	69.0	0.2	1.5	13.3	94	87	51	81.2
8	493.0	261.0	8.0	52.9	34.4	1.3	63.6	35.1	0.0	82.1	58.2	4.4	13.1	6.4	94	94	74	43.4
9	489.0	439.5	4.5	89.9	27.7	17.3	26.1	56.7	0.0	52.2	19.9	1.5	3.1	27.6	95	95	83	44.8
10	490.0	374.5	19.0	76.4	18.3	12.3	19.5	64.2	4.0	22.8	10.9	0.8	0.6	10.4	93	93	80	16.0
11	411.0	357.0	11.5	86.9	11.4	29.1	14.4	47.5	9.0	9.9	0.0	1.3	0.0	8.6	99	98	94	2.4
12	455.0	439.5	9.0	96.6	43.8	18.1	12.3	64.8	4.8	45.9	28.7	1.2	11.9	4.1	96	92	68	48.9
13	525.0	506.0	0.5	96.4	61.5	21.2	17.9	60.9	0.0	66.9	41.1	0.5	24.9	0.4	93	89	68	61.9
14	503.0	402.5	26.5	80.0	12.2	32.8	33.9	33.2	0.1	12.4	1.9	1.5	0.0	8.9	96	96	87	5.3
15	450.0	426.5	7.5	94.8	7.7	15.4	15.5	68.9	0.2	15.6	4.2	3.1	1.0	7.3	93	93	80	13.2
16	537.0	469.5	32.5	87.4	65.3	11.8	40.0	48.0	0.1	84.1	40.0	7.8	25.6	10.7	94	91	66	73.8
17	487.0	434.5	20.5	89.2	16.3	21.5	12.8	49.7	16.0	16.2	0.6	1.4	0.0	14.2	96	95	87	7.2
18	439.0	409.0	7.5	93.2	67.2	14.5	53.7	31.8	0.0	78.3	75.0	0.4	0.6	2.3	88	82	47	75.0
19	552.0	430.5	8.0	78.0	59.5	8.2	65.5	26.2	0.0	68.0	15.1	16.3	4.9	31.8	96	95	76	59.6
20	522.0	278.5	33.0	53.4	80.4	6.6	50.4	40.6	2.3	75.8	0.0	0.4	0.0	75.4	97	96	83	22.0
21	624.0	406.5	38.5	65.1	31.3	15.9	18.2	55.4	10.6	33.1	18.0	0.1	0.0	14.9	95	94	83	19.4
22	492.0	405.5	3.0	82.4	42.9	10.0	35.6	47.3	7.0	63.0	24.4	12.3	4.3	22.0	96	95	85	51.5
23	481.0	423.0	1.0	87.9	21.6	14.5	24.3	61.1	0.0	19.7	2.1	0.0	0.0	17.6	95	94	79	17.1
24	567.0	545.0	4.5	96.1	9.6	16.0	9.0	48.1	27.0	10.5	1.8	0.3	0.0	8.4	97	96	85	7.7
25	551.0	487.5	23.5	88.5	55.5	11.4	55.5	33.1	0.0	66.8	32.4	3.9	4.2	26.3	96	96	90	32.5
26	457.0	393.5	33.0	86.1	49.3	13.9	34.9	34.9	16.3	56.1	39.8	0.2	1.1	15.1	97	95	60	43.4
27	543.0	460.0	10.5	84.7	7.7	18.7	7.9	48.5	24.9	10.3	3.3	0.0	0.0	7.0	97	96	80	7.4
28	545.0	346.0	4.5	63.5	33.5	16.6	26.2	44.8	12.4	33.6	0.2	0.0	0.0	33.5	93	92	85	17.2
29	478.0	354.5	24.5	74.2	32.0	11.8	11.0	69.8	7.3	36.1	13.4	0.0	0.2	22.5	94	93	74	32.8
30	492.0	438.5	6.5	89.1	81.8	11.1	7.6	77.8	3.5	91.9	55.3	3.4	8.5	24.8	91	89	40	83.8
31	522.0	416.5	13.0	79.8	48.8	13.2	56.8	24.4	5.6	57.2	20.0	6.6	10.1	20.5	98	90	66	52.6
32	502.0	345.5	9.5	68.8	16.8	20.3	20.8	52.7	6.2	26.0	0.2	0.2	0.0	25.7	94	94	81	15.8
33	465.0	260.0	21.0	55.9	10.2	12.1	19.4	67.9	0.6	35.3	5.8	3.2	0.2	26.1	95	95	81	18.9
34	394.0	369.5	3.5	93.8	33.6	24.4	19.5	45.3	10.8	41.1	30.4	0.8	2.6	7.3	96	96	60	28.7
35	537.0	440.0	9.5	81.9	21.1	35.5	19.5	36.4	8.6	31.0	17.5	0.7	1.6	11.2	96	96	73	22.1
36	535.0	489.0	0.0	91.4	85.6	20.7	31.9	47.4	0.0	91.4	66.1	5.0	18.4	1.8	95	92	70	87.1
37	357.0	276.5	18.0	77.5	20.6	19.0	26.6	52.1	2.4	34.7	9.8	1.7	0.9	22.4	96	94	81	22.0
38	424.0	379.5	0.0	89.5	19.6	27.4	14.1	54.8	3.7	15.5	3.5	0.2	0.2	11.7	96	96	87	8.6
39	474.0	402.5	8.5	84.9	46.8	16.0	19.8	64.2	0.0	48.3	23.4	0.7	1.0	23.1	98	98	87	26.5
40	504.0	426.0	17.0	84.5	27.2	12.0	31.0	52.3	4.7	35.1	7.2	5.8	2.1	20.0	93	92	77	21.8
41	480.0	381.0	16.0	79.4	27.6	11.5	46.3	41.3	0.8	38.7	17.3	2.4	4.3	14.8	97	97	81	25.0
42	560.0	338.5	12.5	60.4	38.5	26.4	19.2	36.6	17.7	46.6	40.9	0.4	0.0	5.3	96	92	73	46.1
43	561.0	354.0	24.0	63.1	26.1	9.2	40.8	46.5	3.5	45.4	16.4	5.3	4.9	18.8	95	94	68	27.9
44	455.0	436.5	5.5	95.9	50.6	14.2	15.5	66.9	3.4	59.2	29.4	14.0	10.4	5.4	97	95	74	54.5
45	510.0	468.0	6.5	91.8	47.6	9.7	26.0	62.1	2.2	49.5	6.2	3.1	1.4	38.8	96	94	76	37.4
46	502.0	326.5	1.0	65.0	10.9	21.6	13.5	62.6	2.3	30.7	12.5	0.4	0.2	17.6	98	97	81	16.0
47	512.0	368.5	14.0	72.0	11.3	4.3	22.5	73.1	0.0	7.0	0.3	0.8	0.0	5.9	95	95	89	2.6

48	505.0	480.5	0.0	95.1	40.2	16.8	28.1	53.7	1.5	47.4	19.7	2.7	4.0	20.9	96	96	61	24.7
49	444.0	271.5	10.5	61.1	54.1	7.9	82.1	9.9	0.0	79.6	53.3	8.4	14.6	3.3	95	94	68	47.7
50	484.0	465.0	4.5	96.1	63.2	16.8	22.3	61.0	0.0	66.6	39.5	1.4	24.0	1.7	96	87	34	61.4
51	441.0	408.5	4.5	92.6	12.5	20.6	15.9	56.7	6.9	10.7	0.9	0.0	0.3	9.5	98	97	80	7.2
52	484.0	403.5	33.5	83.4	18.3	16.7	40.4	36.4	6.4	16.7	2.4	2.5	0.4	11.3	97	97	80	10.7
53	515.0	432.0	65.5	83.9	26.9	17.8	21.6	50.2	10.3	26.5	3.8	0.6	0.1	22.1	98	96	87	16.3
54	452.0	381.5	14.5	84.4	51.9	19.9	20.1	44.6	15.5	62.7	43.4	1.7	3.0	14.6	97	93	67	51.4
55	507.0	425.0	44.5	83.8	20.6	23.5	46.8	28.5	1.2	57.7	56.9	0.0	0.3	0.6	98	98	86	12.5
56	477.0	455.0	9.0	95.4	8.2	11.9	12.1	64.5	11.5	19.4	8.8	0.0	0.3	10.3	95	94	83	17.7
57	486.0	421.5	26.0	86.7	27.3	20.0	26.7	48.5	4.7	35.3	17.2	0.4	1.1	16.5	94	93	82	26.4
58	370.0	273.5	4.5	73.9	24.2	27.2	6.4	50.5	15.9	30.9	16.0	0.4	0.0	14.5	96	96	91	4.7
59	545.0	460.0	4.5	84.4	31.6	14.6	56.1	27.8	1.5	62.9	24.1	11.5	8.2	19.0	95	95	88	9.0
60	421.0	394.0	6.0	93.6	57.9	27.4	29.2	43.4	0.0	63.0	55.4	1.2	6.7	0.0	95	92	59	53.3
61	527.0	499.0	7.0	94.7	71.2	20.5	44.5	35.0	0.0	76.8	52.3	2.8	21.0	0.7	93	86	61	66.4
62	451.0	445.5	0.5	98.8	46.1	7.5	40.6	42.9	9.0	54.8	39.1	2.2	5.4	8.2	94	94	76	49.8
63	514.0	374.0	19.5	72.8	12.4	14.8	31.0	46.9	7.2	20.9	1.3	2.4	0.2	17.0	98	97	81	9.9
64	518.0	399.5	30.5	77.1	13.8	16.0	10.0	55.2	18.8	16.7	8.9	0.0	0.0	7.8	96	95	83	8.2
65	464.0	377.5	1.0	81.4	27.4	12.8	33.0	36.0	18.1	38.8	8.3	6.8	7.5	16.2	95	95	85	26.4
66	464.0	422.0	17.5	90.9	26.8	24.3	20.9	48.6	6.3	26.7	10.8	3.0	6.7	6.3	94	94	74	18.6
67	510.0	253.5	39.0	49.7	32.5	2.0	49.9	48.1	0.0	64.5	21.1	4.5	18.0	20.8	96	96	84	23.2
68	530.0	433.5	31.5	81.8	24.9	20.8	13.3	65.9	0.1	30.4	9.6	7.1	4.6	9.3	97	97	82	23.3
69	523.0	375.0	26.5	71.7	25.0	22.1	27.1	50.8	0.0	37.1	27.7	4.2	3.7	1.6	94	94	86	13.1
70	512.0	493.5	5.5	96.4	78.3	13.0	37.4	49.6	0.0	84.0	78.9	3.4	0.0	1.7	91	83	42	86.1
71	388.0	358.0	1.0	92.3	45.6	18.6	31.0	50.4	0.0	57.0	32.2	1.3	17.6	6.2	94	93	75	53.9
72	488.0	399.5	18.0	81.9	28.1	2.8	19.5	75.3	2.4	25.2	8.1	0.2	0.0	17.0	94	94	85	18.8
73	530.0	346.5	141.5	65.4	37.2	20.5	21.2	45.0	13.3	33.1	9.5	0.9	2.1	20.6	96	95	86	17.6
74	517.0	393.5	28.5	76.1	13.4	20.5	27.7	41.3	10.5	18.8	6.3	1.1	1.7	9.8	97	96	85	10.3
75	493.0	430.5	18.5	87.3	77.6	17.3	27.5	54.6	0.6	91.8	67.3	2.5	15.1	7.0	97	94	63	66.8
76	454.0	243.0	11.0	53.5	28.0	8.6	20.2	38.9	32.3	44.4	0.2	1.7	0.0	42.5	96	96	87	22.1
77	485.0	418.5	5.5	86.3	31.4	8.6	30.0	57.0	4.4	31.4	16.8	4.9	5.0	4.7	96	96	91	14.1
78	450.0	392.5	32.5	87.2	36.2	7.6	7.9	83.6	0.9	33.2	17.7	0.2	0.8	14.5	97	96	86	22.3
79	422.0	398.0	1.0	94.3	65.1	27.6	14.4	57.9	0.0	72.1	36.2	2.0	28.5	5.4	95	90	51	66.4
80	498.0	257.0	24.5	51.6	13.6	11.9	37.4	43.8	7.0	19.8	6.8	0.0	0.0	13.1	92	92	80	8.7
81	505.0	432.0	22.5	85.5	54.1	20.7	32.2	46.2	0.9	70.0	31.8	6.4	15.4	16.4	94	90	63	60.7
82	459.0	425.0	11.0	92.6	24.9	24.1	10.2	41.9	23.8	30.9	7.6	0.1	0.7	22.4	97	97	76	22.0
83	577.0	436.5	115.5	75.6	72.6	10.7	16.2	73.2	0.0	78.1	50.4	2.5	24.7	0.4	94	90	58	65.2
84	444.0	389.0	6.0	87.6	24.5	11.8	24.2	53.7	10.3	32.9	17.7	0.0	0.0	15.1	94	93	73	25.0
85	476.0	452.0	2.5	95.0	35.0	22.3	16.2	57.1	4.4	47.5	39.4	2.0	4.9	1.2	94	93	54	43.1
86	511.0	444.5	27.5	87.0	14.3	21.5	21.5	54.3	2.7	21.5	1.1	1.3	1.2	17.8	94	94	81	20.0
87	413.0	392.5	9.0	95.0	9.2	17.7	9.0	55.5	17.7	6.1	1.1	0.6	0.0	4.4	94	94	77	6.0
88	515.0	446.0	9.0	86.6	22.4	17.9	7.5	71.6	2.9	44.0	26.5	1.1	3.6	12.8	96	96	64	32.8
89	508.0	484.0	9.5	95.3	7.9	22.4	7.2	58.8	11.6	6.6	0.1	0.4	0.0	6.1	96	96	85	2.8
90	544.0	359.0	35.0	66.0	16.2	11.7	24.0	55.2	9.2	14.0	2.3	3.7	0.3	7.7	96	96	93	2.4
91	620.0	499.0	33.0	80.5	15.4	17.8	11.3	68.6	2.2	26.7	0.7	0.0	0.0	26.0	95	94	82	19.0
92	434.0	394.5	8.0	90.9	9.3	28.4	12.2	44.5	15.0	18.7	7.5	0.8	0.5	10.0	98	98	88	8.7
93	535.0	359.0	23.5	67.1	16.5	15.3	18.1	66.4	0.1	29.2	7.0	0.8	0.5	20.9	95	95	77	15.8
94	506.0	327.0	39.0	64.6	4.8	19.0	12.5	61.5	7.0	8.1	2.6	0.0	0.0	5.5	94	94	80	8.3
95	501.0	482.5	7.5	96.3	32.5	17.9	22.9	52.1	7.0	32.0	4.4	6.0	1.7	19.9	94	94	80	26.4
96	558.0	367.5	8.5	65.9	14.4	14.3	16.3	66.7	2.7	16.2	0.0	1.3	0.0	14.9	95	95	88	7.6
97	505.0	308.5	2.5	61.1	32.2	4.2	21.9	73.9	0.0	55.6	34.8	0.0	0.0	20.8	96	93	60	36.7

98	441.0	405.5	0.0	92.0	27.2	12.3	14.3	48.3	25.0	56.5	32.6	4.9	1.9	17.2	95	95	77	55.7
99	480.0	397.5	25.0	82.8	17.0	19.5	29.2	42.1	9.2	14.8	0.2	0.0	0.0	14.6	95	94	89	5.4
100	419.0	397.5	2.0	94.9	32.2	17.2	25.8	41.4	15.6	41.7	24.5	2.7	0.6	13.9	96	95	60	37.0
101	536.0	392.5	0.0	73.2	8.0	25.6	9.0	49.0	16.3	10.7	0.9	0.2	0.0	9.6	97	96	87	5.6
102	534.0	295.5	28.0	55.3	10.0	20.1	20.6	47.0	12.2	24.0	1.0	1.2	0.0	21.7	96	95	83	17.0
103	527.0	290.0	31.0	55.0	11.3	17.4	9.7	61.6	11.4	18.1	2.1	0.4	0.0	15.5	96	95	76	8.2
104	520.0	202.0	50.5	38.8	7.9	39.9	9.7	50.0	0.5	9.5	0.6	0.3	0.0	8.6	96	96	91	1.0
105	480.0	274.0	39.0	57.1	38.3	8.6	38.0	53.5	0.0	55.2	16.6	7.9	29.8	0.9	95	94	78	31.6
106	537.0	475.5	22.5	88.5	67.3	18.8	26.6	54.6	0.0	72.8	42.0	2.6	21.5	6.7	97	97	84	46.1
107	513.0	490.5	4.0	95.6	73.9	11.3	3.6	85.1	0.0	77.4	57.4	1.1	13.1	6.1	94	85	51	74.7
108	433.0	399.0	6.5	92.1	42.2	26.3	8.0	65.7	0.0	57.4	31.7	2.9	15.8	7.1	95	91	68	55.1
109	535.0	322.5	6.0	60.3	59.0	28.7	36.4	31.5	3.4	111.6	40.9	14.3	9.3	47.1	87	88	49	59.5
110	505.0	363.0	35.5	71.9	15.2	15.6	25.6	56.3	2.5	33.2	13.1	4.5	0.8	14.9	96	95	83	20.1
111	463.0	440.5	8.5	95.1	83.0	12.6	13.3	72.1	2.0	108.7	40.9	0.1	4.8	62.9	93	85	56	104.6
112	532.0	341.5	68.0	64.2	13.3	29.3	18.2	42.6	10.0	19.7	3.9	1.4	2.3	12.1	97	97	86	10.8
113	575.0	373.0	39.0	64.9	24.4	11.1	14.6	68.6	5.6	25.6	11.7	0.5	0.5	12.9	98	97	85	12.0
114	573.0	478.5	28.5	83.5	26.6	18.8	21.1	54.1	6.0	19.1	4.6	2.8	0.8	10.9	95	95	84	12.1
115	483.0	425.5	25.0	88.1	24.8	14.1	14.6	51.7	19.6	35.8	24.4	0.4	0.8	10.2	96	96	84	23.2
116	532.0	345.0	31.5	64.8	12.9	8.8	27.8	43.9	19.4	8.9	0.2	0.2	0.0	8.5	96	96	91	4.9
117	498.0	402.5	28.5	80.8	52.9	18.0	26.1	55.9	0.0	65.4	23.9	4.3	15.8	21.5	95	89	45	43.6
118	511.0	449.0	26.5	87.9	73.8	20.3	22.8	56.9	0.0	98.9	48.4	10.0	31.9	2.5	93	93	80	78.1
119	542.0	372.0	121.0	68.6	31.5	20.4	13.6	55.4	10.6	39.8	15.8	0.6	7.4	16.0	96	96	75	21.8
120	574.0	458.5	2.0	79.9	16.7	28.1	17.3	50.5	4.0	39.0	19.4	1.0	1.4	17.1	96	95	69	35.6
121	490.0	359.5	74.5	73.4	25.4	16.4	10.7	61.3	11.5	28.0	2.8	0.0	0.0	25.2	96	96	87	11.5
122	539.0	506.5	12.5	94.0	54.6	21.9	24.6	53.0	0.5	73.0	44.7	1.1	6.8	20.5	96	94	74	64.9
123	563.0	466.5	0.0	82.9	25.6	18.0	28.3	47.7	6.0	40.5	16.2	2.1	2.7	19.5	95	94	81	32.2
124	589.0	431.5	145.5	73.3	30.7	32.2	11.0	49.5	7.3	37.3	29.1	0.0	1.0	7.2	98	96	87	16.7
125	526.0	463.5	9.5	88.1	48.6	10.2	20.7	69.0	0.0	58.9	35.3	0.3	18.3	5.0	92	91	59	54.4
126	447.0	419.0	4.5	93.7	34.2	17.8	23.4	51.7	7.2	58.6	21.9	0.0	0.1	36.5	93	94	57	56.1
127	550.0	266.0	4.5	48.4	9.6	5.8	28.0	50.4	15.8	21.7	6.1	0.0	0.0	15.6	96	96	75	10.7
Mear	497.5	396.7	19.7	80.2	33.2	16.8	24.8	52.1	6.2	42.5	20.5	2.3	4.8	14.8	95.3	94.0	75.9	30.6
SE	4.3	5.9	2.2	1.2	1.8	0.6	1.3	1.1	0.6	2.2	1.7	0.3	0.7	1.0	0.2	0.3	1.1	2.1
