# Supplementary material for: Treatment of obstructive sleep apnea with a simple CPAP device
Source: Sleep Breath. 2023 May 22;27(6):2351–9. doi: 10.1007/s11325-023-02823-2 (PMC10656318; doi:10.1007/s11325-023-02823-2)
Supplement: Supplementary file 1 — Supplementary file1 (ZIP 1292 KB) [file 11325_2023_2823_MOESM1_ESM.zip › table E-1.pdf]

Subject NO.	Age	Sex	BMI (kg/m^2)	Neck circumference (cm)	Waist circumference (cm)	Hip circumference (cm)	ESS	Duration Hypertension (y)	Duration Coronary hear disease(y)	Duration Stroke(y)	Duration COPD(y)	Duration Athma(y)	Duration Diabetes (y)	Duration Smoking(y)	No. of cigarettes per day	Alcohol (grams/week)	Systolic BP (mmHg)	Diastolic BP (mmHg)
1	75	F	20.3	30.0	75.0	90.0	6	13	10	0	0	0	0	0	0	0	122	63
2	72	M	27.9	38.0	98.0	100.0	7	8	3	14	0	0	0	0	0	0	133	48
3	64	F	28.0	37.0	102.0	99.0	7	40	10	13	0	0	17	0	0	0	150	76
4	38	M	33.6	45.0	116.5	113.0	0	10	0	0	0	0	0	0	0	0	156	106
5	34	M	24.5	37.0	90.0	100.0	2	0	0	0	0	0	0	0	0	0	76	97
6	44	M	27.1	37.0	94.0	96.0	3	7	1	0	0	0	1	20	20	0	106	62
7	46	M	39.8	51.0	129.0	124.0	13	0	0	0	0	0	0	35	5	0	138	83
8	63	M	31.2	45.0	109.0	106.0	8	0	0	0	0	0	0	30	20	0	144	86
9	62	M	30.1	42.0	101.0	109.0	10	20	0	0	0	0	0	40	15	0	150	89
10	56	M	28.7	39.0	103.0	104.0	11	0	0	0	0	0	0	0	0	0	113	73
11	46	M	23.9	39.0	84.0	98.0	6	0	0	0	0	0	0	20	20	0	116	67
12	48	M	22.9	37.0	80.0	90.0	7	10	0	0	0	0	1	0	0	0	142	92
13	44	M	24.9	42.0	86.0	99.0	7	0	0	0	0	0	0	20	40	0	130	90
14	75	M	24.8	38.0	92.0	102.0	3	20	0	27	0	0	0	15	3	0	118	62
15	64	M	25.4	36.0	88.0	93.0	1	12	7	0	0	0	0	0	0	0	126	76
16	49	M	31.5	44.0	113.0	109.0	4	0	0	0	0	0	0	0	0	0	117	71
17	32	M	24.3	38.0	91.0	95.0	0	0	0	0	0	0	0	0	0	100	136	89
18	35	M	37.2	46.5	118.0	112.0	16	0	0	0	0	0	0	14	30	0	117	82
19	78	F	29.5	41.0	102.0	103.0	5	33	14	14	0	0	3	0	0	0	126	73
20	35	F	24.7	32.0	82.0	91.0	7	0	0	0	0	0	0	0	0	0	116	77
21	59	F	25.6	36.0	86.5	89.0	5	0	0	0	0	0	0	0	0	0	135	86
22	76	F	31.6	38.0	106.0	105.0	5	10	3	0	0	0	20	0	0	0	127	75
23	71	F	28.1	35.5	105.0	100.0	0	20	15	0	0	0	10	0	0	0	143	88
24	51	F	20.5	32.0	79.0	88.0	9	1	0	0	0	0	0	0	0	0	130	72
25	47	M	26.1	43.0	98.0	102.0	13	3	0	0	0	0	0	30	20	0	129	89
26	39	M	31.7	41.0	117.0	113.0	0	3	0	0	0	0	0	8	24	0	147	91
27	62	F	22.8	34.5	86.0	90.0	5	10	0	0	0	0	0	0	0	0	173	103
28	52	M	25.4	37.5	93.0	97.0	7	0	0	0	1	0	0	25	40	0	137	85
29	74	F	31.2	37.0	109.0	106.0	3	20	8	8	0	0	6	0	0	0	127	83
30	48	M	37.9	46.0	126.0	128.0	12	1	0	0	0	0	0	0	0	200	134	86
31	57	M	28.6	41.0	98.0	106.0	10	15	4	1	0	0	0	0	0	0	146	81

32	68	M	22.2	35.0	80.0	85.0	6	0	0	0	0	0	0	0	0	0	130	81
33	75	M	25.9	40.0	101.0	106.0	5	20	8	8	0	0	0	0	0	0	143	76
34	31	M	26.7	40.0	95.0	108.0	3	13	0	0	0	0	0	4	10	0	139	91
35	48	M	25.7	37.0	91.0	103.0	0	0	0	0	0	0	0	0	0	0	135	97
36	34	M	30.1	43.0	102.0	109.0	6	0	0	0	0	0	0	1	20	0	111	80
37	47	M	24.0	38.0	85.0	95.0	13	1	1	0	0	0	0	0	0	0	137	91
38	53	M	24.2	36.0	90.0	101.0	13	0	0	0	0	0	0	0	0	0	104	60
39	57	M	32.7	41.0	110.0	113.0	19	12	0	0	0	0	10	40	20	400	129	74
40	57	M	26.1	39.0	109.0	106.0	4	7	0	0	1	0	0	10	40	100	143	77
41	53	M	29.4	43.0	97.5	101.0	5	25	0	0	0	0	6	0	0	0	143	87
42	26	M	24.9	38.0	95.0	102.0	13	0	0	0	0	0	0	0	0	0	128	79
43	72	F	27.0	34.0	101.0	106.0	21	7	0	1	0	0	0	0	0	0	132	81
44	60	M	29.7	41.0	102.0	106.0	0	0	20	0	0	0	0	20	30	0	143	65
45	42	M	27.9	39.0	103.0	101.0	9	0	0	0	0	0	0	15	20	0	132	73
46	50	F	23.6	32.0	92.0	95.0	5	0	0	0	0	0	0	0	0	0	116	67
47	60	M	25.0	42.0	112.0	110.0	0	0	0	0	0	0	0	0	0	0	126	65
48	41	M	27.9	43.0	95.0	105.0	6	0	0	0	0	0	0	0	0	0	125	86
49	71	M	33.6	43.0	116.0	119.0	14	18	0	0	0	0	0	20	40	0	178	92
50	44	M	28.0	38.0	98.0	102.0	3	1	0	0	0	0	0	10	20	0	131	86
51	21	M	21.0	34.5	94.5	86.5	13	0	0	0	0	0	0	10	5	0	112	71
52	47	M	27.6	39.0	99.0	98.0	12	0	0	0	0	0	0	0	0	0	142	91
53	21	M	21.5	36.0	82.0	92.0	4	0	0	0	0	0	0	0	40	0	112	56
54	36	M	24.7	36.0	92.0	101.0	7	0	0	0	0	0	0	0	0	0	137	84
55	16	M	21.7	36.4	83.5	100.0	6	0	0	0	0	0	0	0	0	0	108	71
56	69	F	25.8	34.0	85.0	99.0	2	20	0	10	0	0	0	0	0	0	138	70
57	43	M	34.4	42.0	108.0	109.0	2	0	0	0	0	0	0	0	0	0	126	68
58	50	M	26.2	39.0	89.0	101.0	9	0	0	0	0	0	0	0	0	0	105	67
59	67	M	24.4	42.0	100.0	100.0	18	20	0	0	2	0	0	60	20	1000	153	16
60	53	M	27.3	40.0	102.0	102.0	12	0	0	0	0	0	0	40	20	250	155	94
61	40	M	30.1	41.0	101.0	105.0	24	0	0	0	0	0	0	4	50	0	166	99
62	47	M	22.0	34.5	82.0	91.0	5	0	0	0	0	0	0	24	30	1000	107	65
63	38	M	28.6	39.0	98.0	101.0	6	0	0	0	0	0	0	0	0	0	125	80
64	31	M	23.7	36.0	79.0	94.0	0	0	0	0	0	0	0	0	0	0	109	71
65	61	M	24.5	39.0	89.0	97.0	5	0	0	0	0	0	0	40	20	0	114	77
66	47	M	25.5	37.0	92.0	90.0	7	0	0	0	0	0	0	0	0	0	126	77
67	52	M	27.4	38.0	99.5	102.0	2	3	0	0	0	0	0	30	40	500	127	81

68	41	M	28.2	38.5	93.0	96.0	6	0	0	0	0	0	0	20	20	0	135	78
69	67	M	20.7	35.0	79.0	94.0	3	0	0	0	0	0	0	30	20	0	132	90
70	34	M	36.8	48.0	121.0	120.0	11	1	0	0	0	0	0	10	15	0	166	109
71	48	M	25.0	39.0	87.0	90.0	13	0	0	0	0	0	0	30	20	0	111	71
72	86	M	28.7	42.0	114.0	100.0	12	0	0	0	0	0	0	20	50	7000	102	55
73	31	M	27.4	38.0	90.0	105.0	8	0	0	0	0	0	0	0	0	0	114	62
74	50	M	25.2	38.5	88.5	96.5	0	0	0	0	0	0	0	15	10	0	115	68
75	39	M	29.4	41.5	108.5	110.0	14	0	0	0	0	0	0	9	10	0	131	82
76	49	F	29.7	34.5	97.0	104.5	6	0	0	0	0	0	0	0	0	0	132	85
77	76	M	21.8	34.5	96.5	94.0	1	30	0	0	0	0	0	0	0	0	148	73
78	73	F	23.2	34.5	86.0	85.0	2	21	15	6	0	0	10	0	0	0	127	71
79	57	M	28.3	39.0	99.0	105.0	6	0	0	0	0	0	0	10	10	0	138	92
80	61	M	29.0	39.5	102.0	99.0	12	0	0	0	0	0	0	35	20	0	145	91
81	53	M	32.8	44.0	113.0	108.0	14	0	0	0	0	0	0	0	0	0	117	69
82	28	M	27.4	37.0	95.0	105.0	5	0	0	0	0	0	0	0	0	0	120	70
83	37	M	26.1	38.0	92.5	101.5	6	0	0	0	0	0	0	20	7	0	128	77
84	31	M	26.0	38.0	92.0	102.0	20	0	0	0	0	0	0	10	40	0	141	84
85	49	M	31.1	40.0	104.0	113.0	10	12	0	0	0	0	0	0	0	0	140	80
86	63	M	29.9	40.0	106.0	105.5	9	0	0	0	0	0	0	2	20	0	114	73
87	56	F	27.2	37.0	93.0	98.0	18	0	0	0	0	0	0	0	0	0	106	66
88	73	M	24.1	38.0	92.0	99.0	4	0	0	0	0	0	0	0	0	0	103	66
89	30	M	24.8	38.0	86.0	96.0	0	0	0	0	0	0	0	0	0	0	108	73
90	53	M	20.8	36.0	76.0	92.0	2	0	0	0	0	0	0	0	0	0	126	71
91	51	M	28.5	39.0	99.0	102.0	15	0	0	0	0	0	0	0	0	0	113	68
92	31	M	24.6	37.0	86.0	92.0	4	0	0	0	0	0	0	0	0	0	106	72
93	56	M	25.5	39.0	93.0	99.0	7	0	0	0	0	0	0	12	4	0	131	83
94	65	F	29.3	42.0	100.0	99.0	0	0	0	0	0	0	0	0	0	0	173	83
95	48	M	27.7	38.0	93.0	102.0	6	0	0	0	0	0	0	10	10	0	141	95
96	46	M	25.8	37.0	89.0	95.0	8	0	0	0	0	0	0	0	0	0	124	72
97	51	M	28.7	42.0	106.0	110.0	11	0	0	0	0	0	0	0	0	0	126	69
98	75	F	23.7	32.0	80.0	91.0	0	7	0	0	0	0	0	0	0	0	148	66
99	66	M	32.1	41.5	111.0	111.0	3	9	9	0	0	0	3	40	20	0	130	65
100	43	M	27.2	43.5	109.0	109.0	9	4	0	0	0	0	0	4	10	0	139	90
101	58	F	22.5	31.0	78.0	92.5	5	0	0	0	0	0	0	0	0	0	102	62
102	63	F	32.5	37.0	111.0	102.0	4	0	0	0	0	0	0	0	0	0	140	67
103	48	M	29.9	40.0	100.0	107.0	4	0	0	0	0	0	0	20	20	0	143	76

104	60	M	22.1	34.5	80.0	89.0	8	0	0	0	0	0	0	15	10	0	138	86
105	69	M	24.0	34.5	87.5	96.5	7	0	0	0	0	0	0	16	30	0	114	49
106	50	M	23.1	37.5	89.0	93.0	15	0	0	0	0	0	0	25	40	0	105	68
107	38	M	29.7	38.0	94.0	101.0	7	0	0	0	0	1	0	0	0	0	121	81
108	32	M	29.3	39.5	99.0	100.0	2	0	0	0	0	0	0	0	0	500	116	71
109	48	M	28.6	41.0	94.0	101.0	8	0	0	0	0	0	0	20	20	0	120	85
110	75	F	27.4	31.0	84.0	96.0	3	20	0	15	0	0	10	0	0	0	144	68
111	24	M	32.0	41.5	107.5	100.0	3	0	0	0	0	0	0	0	0	0	139	80
112	47	F	31.0	36.0	101.0	108.0	6	0	0	0	0	0	0	0	0	0	121	73
113	51	M	28.0	40.0	96.0	103.0	5	0	0	0	0	0	0	20	20	0	120	78
114	57	M	26.8	40.0	95.5	99.0	5	0	5	0	0	0	0	15	20	0	144	85
115	63	F	25.2	38.0	95.0	106.0	9	0	0	0	0	0	14	0	0	0	136	67
116	29	M	26.1	39.0	94.0	104.0	1	0	0	0	0	0	0	0	0	0	129	74
117	64	M	26.9	38.0	100.0	105.0	11	10	0	0	0	0	0	0	0	0	128	83
118	57	M	25.7	40.0	100.0	105.0	5	5	0	0	0	0	0	20	40	0	147	63
119	45	M	28.8	44.0	96.0	105.0	19	10	0	0	0	0	0	15	20	0	143	81
120	56	M	25.4	38.0	93.0	92.5	10	0	0	0	0	0	0	0	0	0	129	85
121	32	M	25.7	39.0	93.0	99.0	9	1	0	0	0	0	0	10	6	0	129	74
122	24	M	31.1	43.0	103.0	114.0	13	0	0	0	0	0	0	6	18	200	115	75
123	55	M	31.4	42.0	111.0	108.0	7	8	0	0	0	0	0	10	20	0	138	86
124	39	M	20.5	36.0	87.5	102.0	0	0	0	0	0	0	0	10	10	0	130	84
125	55	M	29.6	40.0	100.0	114.0	16	0	0	0	0	0	0	8	20	0	118	76
126	51	M	27.1	37.0	88.0	94.0	6	4	0	0	0	0	0	30	40	0	134	86
127	51	M	26.0	34.0	96.0	103.0	16	0	0	0	0	0	0	15	10	0	129	70
