# Supplementary figures and images for: Treatment of obstructive sleep apnea with a simple CPAP device
Source: Sleep Breath. 2023 May 22;27(6):2351–9. doi: 10.1007/s11325-023-02823-2 (PMC10656318; doi:10.1007/s11325-023-02823-2)

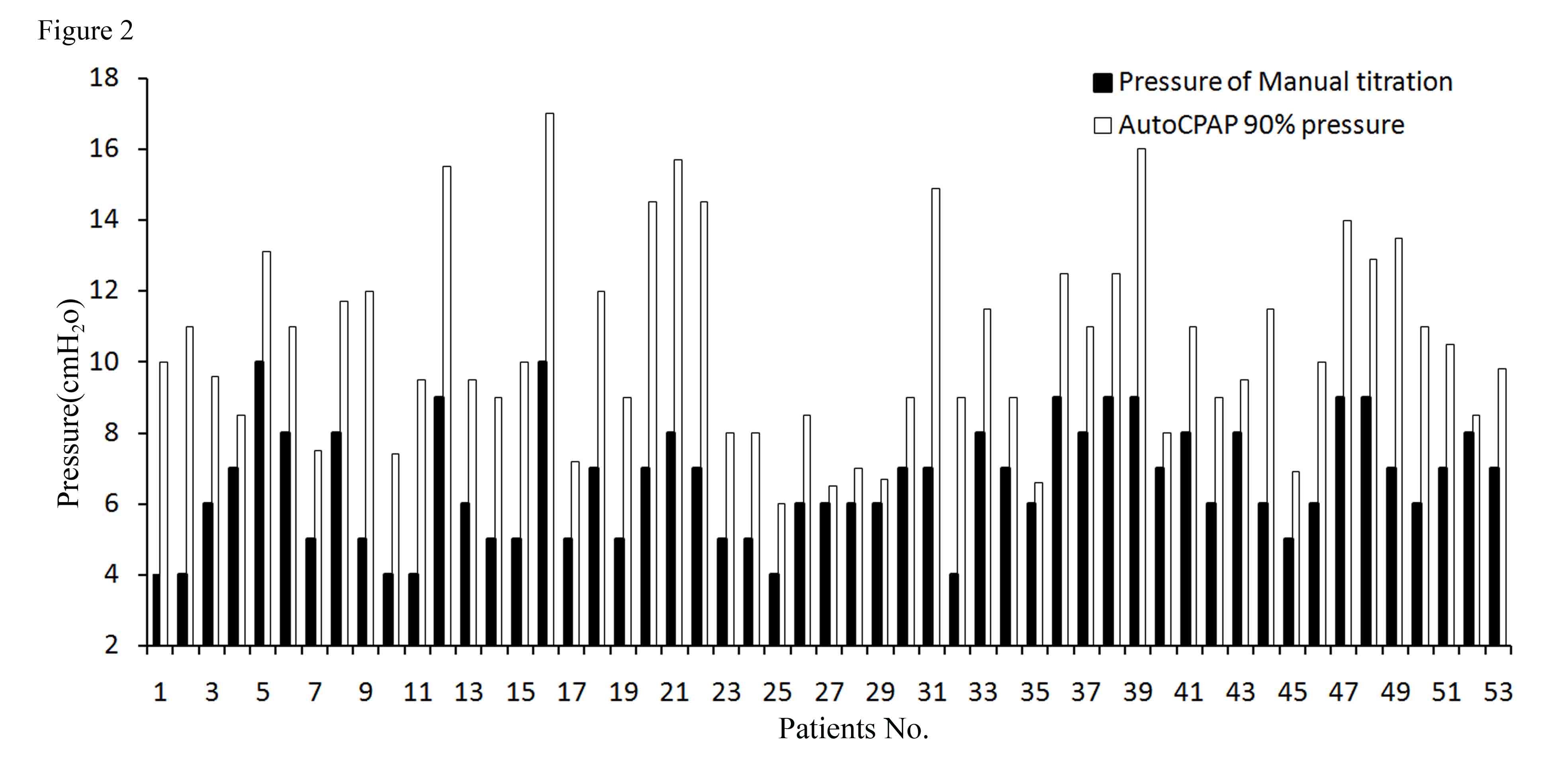

Supplement: Supplementary file 1 — Supplementary file1 (ZIP 1292 KB) [file 11325_2023_2823_MOESM1_ESM.zip › Figure 2.jpg]
